# Repression of a Potassium Channel by Nuclear Hormone Receptor and TGF-β Signaling Modulates Insulin Signaling in *Caenorhabditis elegans*

**DOI:** 10.1371/journal.pgen.1002519

**Published:** 2012-02-16

**Authors:** Donha Park, Karen L. Jones, Hyojin Lee, Terrance P. Snutch, Stefan Taubert, Donald L. Riddle

**Affiliations:** 1 Centre for Molecular Medicine and Therapeutics, University of British Columbia, Vancouver, Canada; 2 Michael Smith Laboratories, University of British Columbia, Vancouver, Canada; 3 Department of Medical Genetics, University of British Columbia, Vancouver, Canada; 4 Department of Biochemistry, College of Science, Yonsei University, Seoul, Korea; Stanford University School of Medicine, United States of America

## Abstract

Transforming growth factor β (TGF-β) signaling acts through Smad proteins to play fundamental roles in cell proliferation, differentiation, apoptosis, and metabolism. The Receptor associated Smads (R-Smads) interact with DNA and other nuclear proteins to regulate target gene transcription. Here, we demonstrate that the *Caenorhabditis elegans* R-Smad DAF-8 partners with the nuclear hormone receptor NHR-69, a *C. elegans* ortholog of mammalian hepatocyte nuclear factor 4α HNF4α), to repress the *exp-2* potassium channel gene and increase insulin secretion. We find that NHR-69 associates with DAF-8 both *in vivo* and *in vitro*. Functionally, *daf-8 nhr-69* double mutants show defects in neuropeptide secretion and phenotypes consistent with reduced insulin signaling such as increased expression of the *sod-3* and *gst-10* genes and a longer life span. Expression of the *exp-2* gene, encoding a voltage-gated potassium channel, is synergistically increased in *daf-8 nhr-69* mutants compared to single mutants and wild-type worms. In turn, *exp-2* acts selectively in the ASI neurons to repress the secretion of the insulin-like peptide DAF-28. Importantly, *exp-2* mutation shortens the long life span of *daf-8 nhr-69* double mutants, demonstrating that *exp-2* is required downstream of DAF-8 and NHR-69. Finally, animals over-expressing NHR-69 specifically in DAF-28–secreting ASI neurons exhibit a lethargic, hypoglycemic phenotype that is rescued by exogenous glucose. We propose a model whereby DAF-8/R-Smad and NHR-69 negatively regulate the transcription of *exp-2* to promote neuronal DAF-28 secretion, thus demonstrating a physiological crosstalk between TGF-β and HNF4α-like signaling in *C. elegans*. NHR-69 and DAF-8 dependent regulation of *exp-2* and DAF-28 also provides a novel molecular mechanism that contributes to the previously recognized link between insulin and TGF-β signaling in *C. elegans*.

## Author Summary

All animals must ensure metabolic homeostasis; if they fail to do so, diseases such as obesity and diabetes can develop. To maintain glucose balance, insulin is secreted upon glucose intake in a highly regulated and coordinated process. Previous studies suggested that the transforming growth factor beta (TGF-β) signaling pathway regulates insulin secretion in mammals. In the genetically tractable roundworm *Caenorhabditis elegans*, TGF-β and insulin signaling modulate larval development and aging, although the molecular link between insulin and TGF-β signaling remains poorly understood. In this study, we show that the TGF-β signaling component DAF-8 partners with NHR-69, a nuclear hormone receptor, to control the expression of the potassium channel *exp-2*, which in turn modulates the secretion of an insulin-like peptide. A loss-of-function *exp-2* mutant exhibits increased insulin secretion and a shortened life span, whereas a gain-of-function mutant exhibits decreased insulin secretion. We also show that tissue-specific expression of *nhr-69* in a pair of neurons that secrete neuropeptides causes reduced glucose content, increased insulin-like peptide levels and a lethargic phenotype. Because insulin and TGF-β signaling are linked to numerous diseases, our data may provide novel insights into the mechanisms contributing to pathophysiological changes.

## Introduction

Transforming growth factor β (TGF-β) signaling plays fundamental roles in cell proliferation, differentiation and apoptosis [1]. TGF-β binding induces the formation of a heterotetrameric type I and type II transmembrane receptor complex, and the type I receptor then phosphorylates the C-terminal Ser-X-Ser motif of receptor-associated Smad (R-Smad) transcription factors [2]. Phosphorylated R-Smads form homodimers and heterotrimers with a Co-Smad and translocate to the nucleus where they regulate the transcription of target genes [1].

TGF-β signaling is evolutionarily conserved; for example, in the lower metazoan *Caenorhabditis elegans*, two partially overlapping TGF-β signaling pathways have been described. One controls body size and male reproductive development whereas the other controls the formation of a specialized larval stage, the dauer [3]–[5]. The dauer larva is a developmentally arrested, long-lived and stress resistant diapause stage, formation of which is induced by overcrowding, starvation, and high temperatures [5]. Screening for mutations that result either in constitutive (Daf-c) or defective (Daf-d) dauer development [5] revealed multiple signaling circuits that control the dauer developmental switch, including a classical TGF-β signaling pathway. The TGF-β ligand DAF-7 promotes larval growth and inhibits dauer arrest; accordingly, transcription of *daf-7*, which is expressed in the amphid single ciliated neuron I (ASI) chemosensory neurons, is repressed by the dauer-inducing pheromone [6]. Downstream of *daf-7*, *daf-1* and *daf-4* encode the nematode type I and type II TGF-β receptor kinases, respectively, and both genes are required for non-dauer larval development. DAF-1 and DAF-4 phosphorylate DAF-8, an R-Smad that antagonizes the Co-Smad DAF-3 to promote larval growth [7]. In line with these roles, *daf-1*, *daf-4*, *daf-7*, and *daf-8* mutants all exhibit Daf-c phenotypes. In an interesting divergence from mammalian TGF-β signaling, the *C. elegans* Co-Smad DAF-3 represses the transcription of the antagonistic upstream genes *daf-7* and *daf-8* by directly binding their promoters, thus creating a positive feedback loop [7].

Dauer formation is also influenced by other sensing and signaling pathways, such as cyclic guanosine monophosphate (cGMP) signaling, steroid receptor action and insulin/insulin-like growth factor 1 (IGF-1) signaling (IIS) [5]. Interestingly, several recent reports indicate that IIS and TGF-β signaling interact in *C. elegans*. In the regulation of fasting/re-feeding induced quiescence, cGMP, insulin/IGF-1, and TGF-β signaling act as parallel activation signals for the protein kinase G (PKG) EGL-4 [8]. All three pathways also affect insulin gene expression, and the gene expression profiles of TGF-β and IIS pathway mutants exhibit a rather broad overlap [9]–[11]. Intriguingly, *daf-7* mutants exhibit increased nuclear localization of the transcription factor DAF-16 [12], a key target of IIS that is also nuclear in worms carrying mutations in the insulin receptor gene *daf-2*. Moreover, the phosphatase PDP-1, which affects TGF-β signaling downstream of *daf-7*, and likely at the level of *daf-8* and *daf-14*, also promotes DAF-16 nuclear localization and insulin gene expression [13]. Thus, PDP-1 and TGF-β signaling may directly promote the expression of insulin peptides, providing one possible mechanistic link between TGF-β signaling and IIS. Accordingly, both the Daf-c and the longevity phenotypes of TGF-β signaling mutants are suppressed at least partially in a *daf-16* mutant [14] [9]. These studies support the notion that TGF-β signaling may in part act upstream of IIS to regulate dauer formation and longevity. However, a simple linear relationship between TGF-β and IIS is unlikely even in the regulation of life span, because the longevity of TGF-β pathway mutants is very modest compared to IIS pathway mutants [9]. In any case, the mechanisms that couple IIS and TGF-β signaling remain poorly defined, and an apparent lack of recognizable Smad-binding sites amongst the genes co-regulated in IIS and TGF-β pathway mutants [9] suggests that these signaling pathways integrate upstream of transcriptional regulation.

Another mechanism that acts downstream of TGF-β signaling in the dauer formation cascade centers on the Nuclear Hormone Receptor (NHR) DAF-12 [15]. DAF-12 is one of 284 *C. elegans* NHRs that are involved in a variety of processes, including embryonic development, dauer formation, regulation of life span, molting, lipid metabolism, xenobiotic metabolism and excretory duct organogenesis [16] [17]. Of the 284 NHRs, most are related to a Hepatocyte Nuclear Factor 4 alpha (HNF4α) related ancestor. Most of these remain poorly characterized, although several HNF4α orthologs regulate lipid metabolism and/or life span [18]–[22]. To date however, DAF-12 remains the only NHR with a defined role in the regulation of dauer formation.

To better define the regulatory network and the transcriptional complexes associated with *C. elegans* DAF-8/R-Smad, we set out to identify its physical interaction partners. We describe a novel physical and functional interaction between DAF-8 and NHR-69 that affects dauer formation, longevity, and the expression of the potassium channel gene *exp-2*, which in turn affects the secretion of the insulin-like peptide DAF-28. Our data indicate that TGF-β signaling and HNF4α-like signaling exhibit regulatory crosstalk to influence the secretion of an insulin-like peptide in *C. elegans*, providing a novel mechanistic link between TGF-β signaling and IIS.

## Results

### NHR-69 and DAF-8 physically interact *in vitro* and *in vivo*

**Figure 1:**
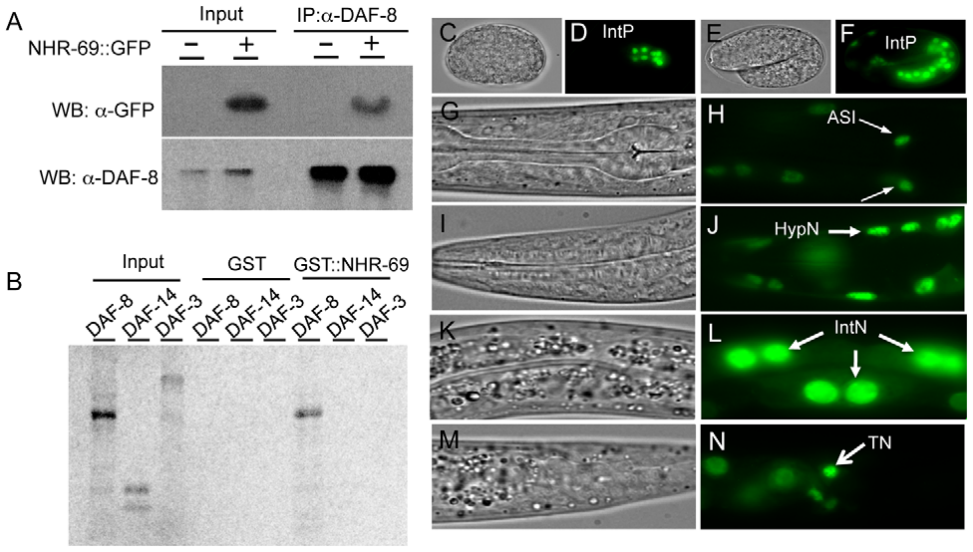
Interaction of NHR-69 with DAF-8 and expression pattern of *nhr-69p::nhr-69::gfp*. (A) *In vivo* co-immunoprecipitation was performed with anti-DAF-8 antibody from wild-type worms and *nhr-69p::nhr-69::gfp* expressing animals, followed by immunoblot with anti-GFP antibody to detect NHR-69::GFP. (B) *In vitro* GST pull-down assay between NHR-69 and SMAD proteins (DAF-8, DAF-14, or DAF-3). C–N show DIC and fluorescence images of developing embryos (C–F) and of adults (G–N) expressing *nhr-69p::nhr-69::gfp.* (C and D) Expression at the E16 stage of intestinal precursor cells in embryos. (E and F) Expression at the comma stage. (G and H) Expression in ASI neurons (arrows). (I and J) Hypodermal expression. (K and L) Intestinal expression. (M and N) Expression in tail neurons. IntP; Intestinal precursor cells, HypN; Hypodermal nucleus, IntN; Intestinal nucleus, TN; Tail neurons.

To identify proteins that bind DAF-8, we used anti-FLAG immunoprecipitation to purify DAF-8 and associated peptides from a worm strain stably expressing a DAF-8::FLAG transgene [7]. Peptide identities were determined by mass spectrometry and we identified several peptides representing NHR-69 (data not shown). NHR-69 is one of the two closest *C. elegans* orthologs of human HNF4α with 39% amino acid identity and 60% similarity (as determined by reciprocal Blast analysis; see also [23]). To validate the interaction between these two transcription factors we generated anti-DAF-8 antibodies (Figure S1) and transgenic animals expressing a translational NHR-69::GFP fusion protein (*nhr-69p::nhr-69::gfp*; see Materials and Methods). Co-immunoprecipitation (Co-IP) showed that NHR-69::GFP associates with DAF-8 *in vivo* (Figure 1A). We also performed *in vitro* binding assays using GST-fusions and *in vitro* transcribed/translated proteins to test for direct interaction between NHR-69 and the functionally distinct SMAD proteins DAF-8, DAF-14, and DAF-3. We found that GST::NHR-69, but not GST alone, associated specifically with DAF-8, but not with DAF-14/R-Smad or DAF-3/Co-Smad (Figure 1B). Together, these data suggest that NHR-69 and DAF-8 specifically interact *in vitro* and *in vivo*.

As DAF-8 and NHR-69 interact physically, we would expect their *in vivo* expression patterns to at least partially overlap. Our previous work showed that DAF-8 is expressed in numerous tissues, including the ASI neurons and the intestine [7]. A NHR-69::GFP fusion protein driven by the *nhr-69* promoter was detected in the nucleus of the E8 intestinal precursor cells in developing embryos (Figure 1D, 1F), and strong intestinal expression persisted throughout larval development until adulthood (Figure 1L). In adults, we also detected expression in the ASI neurons (Figure 1H, Figure S2), in the hypodermis, and in tail neurons (Figure 1J, 1L and 1N). In all of these tissues, NHR-69::GFP was localized exclusively to the nucleus, as expected for a transcription factor. Taken together with our previous findings, NHR-69 and DAF-8 [7] expression overlaps in the ASI neurons and in the intestine, suggesting that these two factors may interact functionally in these two tissues.

### *nhr-69* mutation enhances dauer formation and delays dauer recovery of TGF-β Daf-c mutants

Because DAF-8 and NHR-69 interact physically, we next asked whether NHR-69 affects DAF-8 function. As *nhr-69* RNAi does not cause any obvious phenotypes in wild-type worms [22], we utilized a sensitized genetic background to test whether *nhr-69* contributes to dauer formation. This worm strain, *eri-1(mg366); sdf-9(m708)*, is hypersensitive to RNAi due to the *eri-1* mutation [24], and sensitized to dauer formation due a mutation in the tyrosine phosphatase gene *sdf-9*, which acts parallel to IIS in *C. elegans* [25]. In this background, *nhr-69* RNAi significantly enhanced dauer formation (Figure S3).

To corroborate the RNAi data we obtained a strain carrying a deletion in the *nhr-69* gene, *nhr-69(ok1926)* (see Materials and Methods). The deletion spans the 5′ end of the gene and likely represents a null mutation. In agreement with the RNAi data, the *nhr-69(ok1926)* mutation enhanced the dauer formation of temperature-sensitive *daf-7(e1372)*, *daf-1(m40)*, *daf-8(m85)*, *and daf-14(m77)* mutants at the semi-restrictive temperature (22.5°C Table 1; note that even null mutants in the TGF-β pathway like *daf-8* are temperature-sensitive because dauer formation is temperature-dependent [26]).

**Table 1:** Loss of *nhr-69* enhances the dauer phenotype of TGF-β signaling mutants.

Strain	% dauer formation (N)			
	Single mutants	P value*	Double mutant with *nhr-69*(*ok1926)*	P value**
Wild type^c^	0 (128)	N/A	N/A	
*nhr-69(ok1926)* c	0 (104)	N/A	N/A	
*daf-7(e1372)* a	7.6±2.3 (99)	<0.001	29.5±3.8 (122)	<0.001
*daf-1(m40)* b	60.8±8.7 (127)	<0.001	83.9±5.2 (104)	0.012
*daf-8(m85)* b	62.3±1.7 (136)	<0.001	89.6±2.8 (132)	0.017
*daf-14(m77)* b	78.6±4.5 (118)	<0.001	89.8±5.9 (94)	0.032

Numbers represent percent dauer formation (average ± S.E.M.). N = population size. Three young adults of each genotype were placed on NG agar plates seeded with OP50, then removed after they laid 40–50 eggs. Dauer formation in the F1 generation was scored visually at:

a 20°C,

b 22.5°C, or,

c 25.5°C.

Progeny reaching the L4 stage after 3–4 days (depending on the temperature) were counted and removed. Remaining progeny were scored as dauer larvae.

*Compared to wild type.

**Compared to single mutant.

TGF-β pathway mutants that constitutively arrest as dauer larvae at restrictive temperatures (25°C) also exhibit a temperature-dependent dauer exit phenotype. Thus, we tested whether the *nhr-69(ok1926)* mutation compounded the dauer exit phenotype of TGF-β Daf-c mutants. As expected, TGF-β mutant dauer larvae rapidly recovered from the dauer state, within 24–48 hours after they were transferred to the non-restrictive temperature (15°C; Table 2). In contrast, the *nhr-69(ok1926)* mutation suppressed the dauer exit capacity of *daf-7(e1372)*, *daf-1(m40)*, *daf-8(m85)* and *daf-14(m77)* mutants (Table 2). Hence, loss of *nhr-69* function not only enhances dauer formation, but also delays the dauer exit of TGF-β Daf-c mutants.

**Table 2:** Effect of the *nhr-69* mutation on dauer recovery of TGF-β mutants.

	% recovered from dauer (N)	
Genotype	24 hrs	48 hrs
*daf-7(e1372)*	82.7±4.6 (98)	97.2±2.1 (110)
*daf-1(m40)*	80.2±5.8 (84)	98.7±1.6 (107)
*daf-8(m85)*	85.9±6.3 (94)	97.2±0.9 (93)
*daf-14(m77)*	92.4±3.2 (105)	98.9±0.4 (99)
*daf-7(e1372); nhr-69(ok1926)* a	22.6±6.2 (95)	53.0±3.1 (122)
*daf-1(m40); nhr-69(ok1926)* b	43.6±4.5 (103)	78.9±2.8 (102)
*daf-8(m85) nhr-69(ok1926)* c	34.5±4.8 (100)	67.9±2.5 (94)
*daf-14(m77); nhr-69(ok1926)* d	57.8±3.9 (102)	82.5±7.1 (89)
*daf-8(m85) nhr-69(ok1926); mIs39* e	92.9±1.3 (97)	97.9±1.2 (103)

Numbers are percent dauer recovery (average ± S.E.M.). (N), population size. Three young adults for each genotype were placed on NG agar plates seeded with OP50, then removed after they laid 40–50 eggs. Dauer larvae were induced by the restrictive temperature (25.5°C), and plates were transferred to permissive temperature (15°C) after 72 hrs. The number of L4s and adults recovered from dauer was scored visually at 24 hrs or 48 hrs after temperature shift. *mIs39[nhr-69p::nhr-69::gfp*, *rol-6(su1006)]*.

a Recovery at 24 hrs. Compared to *daf-7(e1372)*: p<0.001.

b Recovery at 24 hrs. Compared to *daf-1(m40)*: p<0.001.

c Recovery at 24 hrs. Compared to *daf-8(m85)*: p<0.001.

d Recovery at 24 hrs. Compared to *daf-14(m77)*: p<0.001.

e Recovery at 24 hrs. Compared to *daf-8(m85)nhr-69(ok1926)*: p<0.001.

### Insulin signaling, but not TGF-β signaling, regulates NHR-69 expression

**Figure 2:**
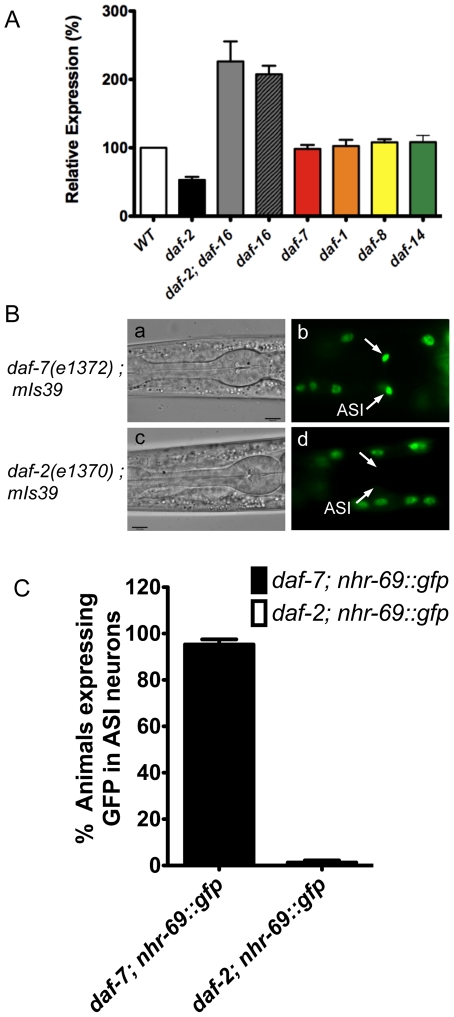
Insulin signaling positively regulates *nhr-69* expression. (A) The bars represent relative levels of *nhr-69* mRNA as determined by qPCR on RNA isolated from *daf-2(e1370)*, *daf-2(e1370)*; *daf-16(mgDf47)*, *daf-16(mgDf47)*, *daf-1(m40)*, *daf-7(e1372)*, *daf-8(m85)* and *daf-14(m77)* mutants (relative to mRNA levels in wild-type worms); mRNA levels were normalized to *act-2*. Error bars indicate standard errors of the mean (SEM) from four biological replicates. (B) Micrographs show DIC and fluorescence images of NHR-69::GFP in *daf-7(e1372)* (a and b) and *daf-2(e1370)* (c and d) mutants. Arrows indicate the ASI neurons. All fluorescence images were taken with identical exposure times. (C) Bar graphs indicate the percentage of animals expressing NHR-69::GFP in ASI neurons in *daf-7(e1372)* (black bar, N = 78) and in *daf-2(e1370)* mutant (white bar, N = 83) shown in (B). Error bars indicate SEM.

Because of the enhanced dauer formation phenotype conveyed by *nhr-69* depletion or mutation, and because TGF-β signaling and insulin signaling both regulate dauer entry and exit [27], we next asked whether these pathways regulate *nhr-69* expression. We used real-time quantitative PCR (qPCR) to quantify *nhr-69* mRNA levels in the TGF-β pathway mutants *daf-7(e1372)*, *daf-1(m40)*, *daf-8(m85)* and *daf-14(m77)*, and in the insulin signaling mutants *daf-2(e1370)*, *daf-16(mgDf47)*, and *daf-2(e1370); daf-16(mgDf47)*. *daf-2* encodes the *C. elegans* insulin/IGF-1 receptor and *daf-16* a downstream transcription factor of the Forkhead Box O family [28] [29]. The mRNA levels of *nhr-69* were similar in wild-type, *daf-1(m40)*, *daf-7(e1372)*, *daf-8(m85)*, and *daf-14(m77)* worms (p>0.05), but were downregulated in *daf-2(e1370)* and upregulated in *daf-2(e1370); daf-16(mgDf47)* and *daf-16(mgDf47)* mutants (Figure 2A), suggesting that DAF-2 regulates *nhr-69 in* DAF-16–dependent fashion.

Given this regulation and given that NHR-69::GFP is expressed in multiple tissues, we next addressed whether insulin or TGF-β signaling affect *nhr-69* expression in any particular tissue. To this end, we generated strains that express the NHR-69::GFP fusion protein in the *daf-2(e1370)* and *daf-7(e1372)* mutant backgrounds. We observed normal NHR-69::GFP expression in the ASI neurons of *daf-7(e1372)* mutants (96±3% of L2 larvae showed GFP fluorescence in both neurons), but NHR-69::GFP was barely detectable in the ASI neurons of *daf-2(e1370)* mutants (0.3±0.04% of L2 larvae showed GFP fluorescence in both neurons, p<0.001; Figure 2B c and d). Together, these data suggest that insulin signaling positively regulates NHR-69 expression specifically in the ASI neurons.

### Insulin signaling is impaired in a *daf-8 nhr-69* double mutant

**Figure 3:**
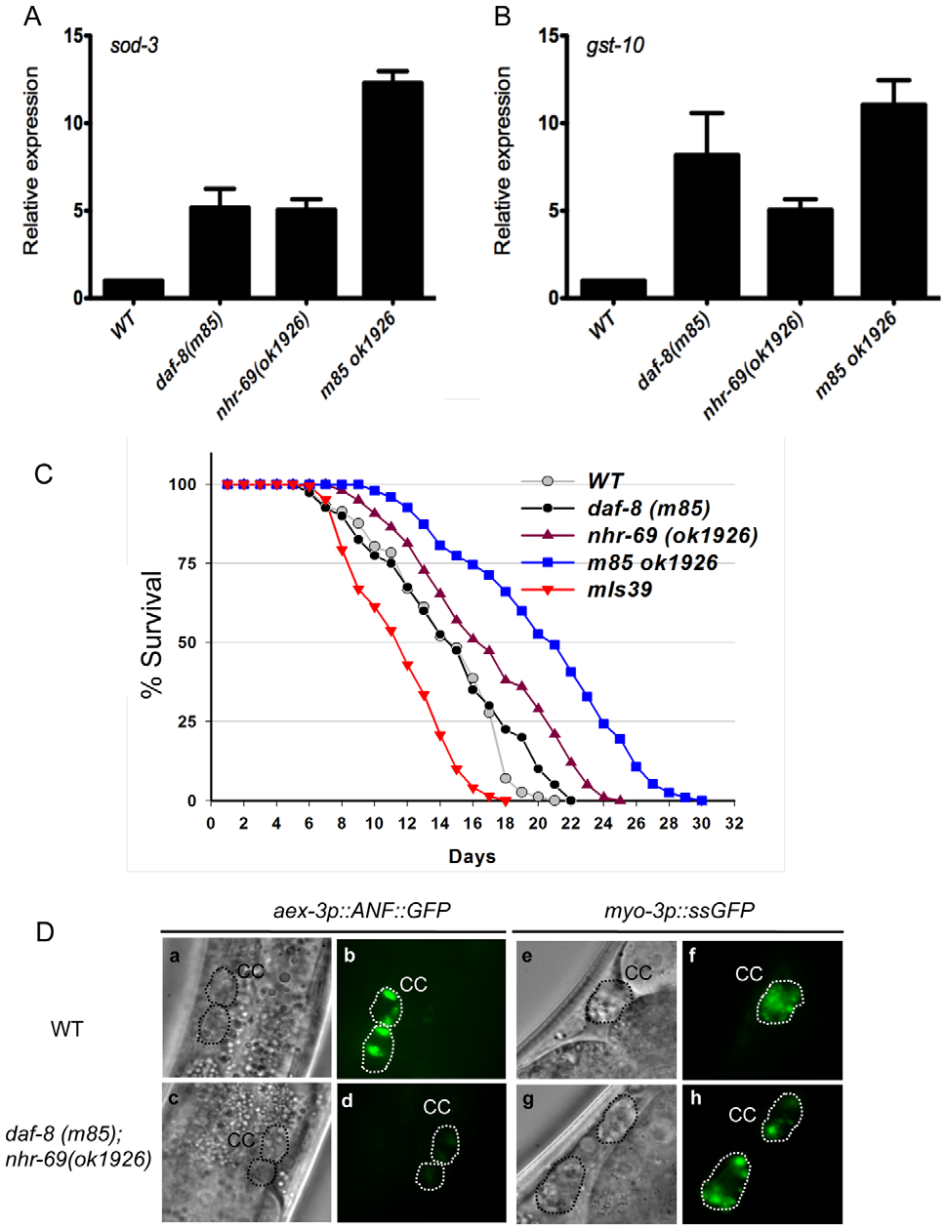
Reduced insulin signaling and neuropeptide secretion in *daf-8 nhr-69* mutants. (A and B) Bar graphs show the relative mRNA levels of *sod-3* and *gst-10* in *daf-8(m85)*, *nhr-69(ok1926)* and *daf-8(m85) nhr-69(ok1926)* mutants relative to wild-type worms, as determined by qPCR (normalized to *act-2*). Age synchronized day-1 adults were used for total RNA extraction. Error bars indicate SEM from three biological replicates. (C) The plots show population survival of wild-type (grey circles), *daf-8(m85)* (black circles), *nhr-69(ok1926)* (purple triangles), *daf-8(m85) nhr-69(ok1926)* (blue squares), and *mIs39[nhr-69p::nhr-69::gfp]* (red inverted triangles) worms at 25.5°C. (D) DIC and fluorescence micrographs show coelomocyte accumulation (CC, dotted circles) of ANF::GFP (expressed from the *oxIs206[aex-3p::ANF::gfp]* transgene; b and d) or ssGFP (expressed from the *arIs37[myo-3P::ssGFP]* transgene; f and h), respectively, in wild-type worms and in *daf-8(m85) nhr-69(ok1926)* mutants, as indicated.

The *daf-8(m85) nhr-69(ok1926)* double mutants exhibited defects in dauer entry and exit, processes regulated by insulin signaling [27]; thus, we next asked whether *daf-8* and *nhr-69* affect insulin signaling in *C. elegans*. First, we examined the expression of *sod-3* and *gst-10*, two downstream targets of IIS, which are regulated by the transcription factors DAF-16 and SKN-1, respectively [30] [31]. SKN-1 is a transcription factor of the bZIP family that acts downstream of *daf-2* in *C. elegans*. qPCR analysis showed that both transcripts were upregulated in the *daf-8(m85)* single mutant; for *sod-3*, this is consistent with previous findings that TGF-β signaling regulates *daf-16* target genes [9]. Furthermore, *sod-3* and *gst-10* induction was enhanced in the *daf-8(m85) nhr-69(ok1926)* double mutant, suggesting that insulin signaling was further reduced in the double mutant (Figure 3A, 3B).

Because adult life span is extended by reduced insulin signaling [28], we next tested whether *nhr-69* and *daf-8* affect longevity. Figure 3C shows that *nhr-69(ok1926)* single mutants exhibited a slightly extended life span relative to wild-type worms (mean life span 17.4±1.3 days, p = 0.027), whereas the *daf-8(m85)* mutant life span was similar to that of wild type (mean life span 14.6±0.8 days, p>0.05; Figure 3C). Notably, the life span of *daf-8(m85) nhr-69(ok1926)* double mutants was significantly longer than that of wild type worms (mean life span 21.5±1.7 days, p<0.001; Figure 3C), consistent with reduced insulin signaling. By contrast, animals over-expressing NHR-69::GFP exhibited a shortened life span (mean life span 11.8±0.6 days, p<0.001) (Figure 3C and Table S1). Together, these data suggest that *daf-8* and *nhr-69* cooperatively affect insulin signaling in *C. elegans*.

### Defective neuropeptide secretion in *daf-8 nhr-69* mutants

Next, we wished to define the mechanism by which *nhr-69* and *daf-8* affect insulin signaling. Given that both genes are expressed in the neuropeptide-secreting ASI neurons, we hypothesized that they might control the release of neuropeptides such as the insulin-like peptide DAF-28, which is secreted by the ASI neurons [32]. To examine this possibility, we first attempted to introduce the DAF-28::GFP transgene into the *daf-8(m85)* mutant background. For unknown reasons, these worms exhibited a non-recoverable Daf-c phenotype, making it impossible to study DAF-28::GFP secretion in this background (data not shown). As an alternative approach, we used worms expressing an *aex-3* promoter-driven ANF::GFP (human Atrial Natriuretic Factor-GFP fusion protein), which can be used as a surrogate to study neuropeptide secretion in *C. elegans* [33]. *aex-3p::ANF::GFP* is expressed in a pan-neuronal fashion, secreted into the inter-organ space (pseudocoelom), and then endocytosed by the coelomocytes, scavenger-like cells that endocytose secreted material [33]. The accumulation of ANF::GFP in coelomocytes was similar in wild-type worms and *daf-8(m85)* and *nhr-69(ok1926)* single mutants, but *daf-8(m85) nhr-69(ok1926)* double mutants accumulated less ANF::GFP (Figure 3D d, Figure S4A). The overall neuronal *ANF::GFP* gene expression was comparable in all worm strains (Figure S4C).

The differential ANF::GFP accumulation in coelomocytes could result either from differences in secretion or endocytosis. Thus, we tested whether the *daf-8(m85) nhr-69(ok1926)* mutants are generally defective for endocytosis. Specifically, we examined a signal sequence GFP (ssGFP) driven by the *myo-3* promoter, which is expressed in muscle cells, secreted into the pseudocoelom and endocytosed by coelomocytes [34]. The accumulation of ssGFP in coelomocytes was similar in wild-type worms and in *daf-8(m85) nhr-69(ok1926)* double mutants (Figure 3D f and h, Figure S4B), suggesting that the *daf-8(m85) nhr-69(ok1926)* mutants are defective specifically for neuropeptide secretion and not for endocytosis.

### NHR-69 directly targets the potassium channel gene *exp-2*

**Figure 4:**
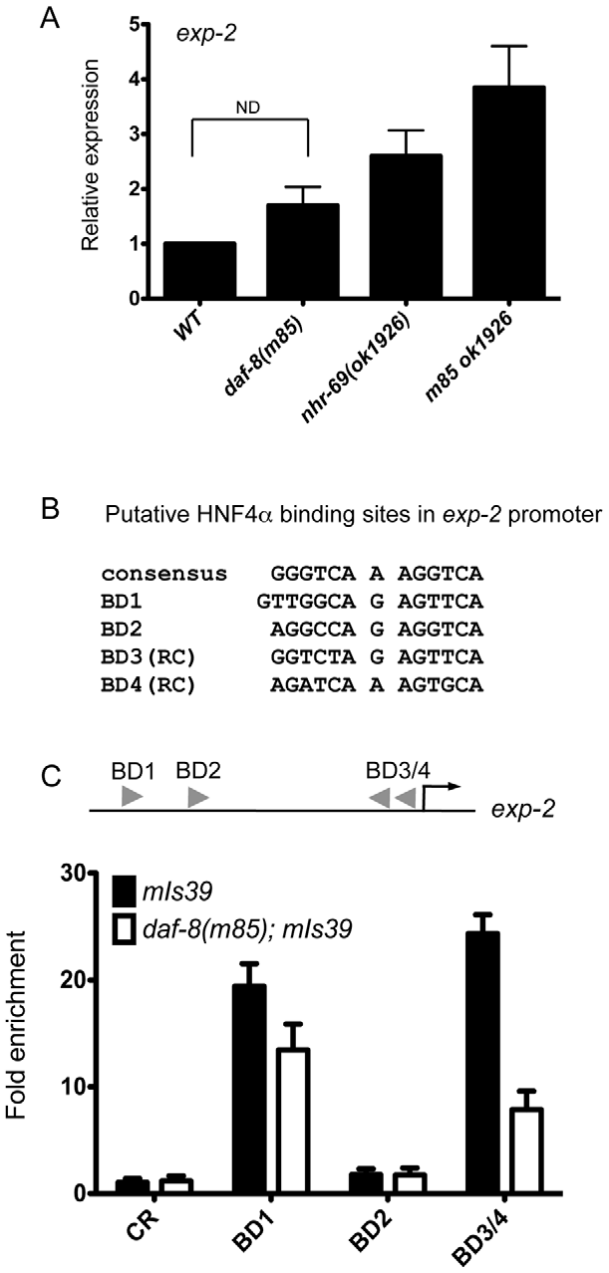
NHR-69 directly regulates *exp-2* transcription. (A) The bar graphs represent the relative levels of *exp-2* mRNA in *daf-89(m85)*, *nhr-69(ok1926)*, and *daf-8(m85) nhr-69(ok1926)* mutants relative to wild type as determined by qPCR (normalized to *act-2*). Error bars indicate SEM from three biological replicates. Age synchronized day-1 adults were used for total RNA extraction. (B) HNF4α binding consensus sequence and putative NHR-69/HNF4α-like binding sites in the *exp-2* promoter region. The positions of possible binding sites 5′ of the ATG of *exp-2* are BD1 (−2342 bp), BD2 (−1832 bp), BD3 (−118 bp) and BD4 (−86 bp), respectively. RC, reverse complement. (C) ChIP-qPCR with anti-GFP antibody reveals that NHR-69 is directly associated with at least two sites in the 5′ regulatory region of *exp-2*. The arrowheads indicate the putative HNF4α binding sites shown in (B). Anti-GFP antibody precipitated sequences from BD1 and BD3 and/or BD4. In a *daf-8* background, binding of NHR-69::GFP was reduced 27% for BD1 and 71% for BD3/BD4. Error bars indicate SEM from three biological replicates.

Although our data suggest that NHR-69 and DAF-8 control neuropeptide secretion, the target genes that might underlie this process remained unknown. Intriguingly, a yeast-one-hybrid (Y1H) study found that NHR-69 binds the promoter of *exp-2*, which encodes a member of the voltage-gated transmembrane potassium channel (Kv) family [35] [36]. Using qPCR, we found that *exp-2* expression was slightly upregulated in the *daf-8(m85)* and *nhr-69(ok1926)* single mutants and synergistically upregulated in the *daf-8(m85) nhr-69(ok1926)* double mutant (Figure 4A).

The Y1H data suggested that NHR-69 can directly bind the *exp-2* promoter. Given the similarity of NHR-69 to mammalian HNF4α, we examined the 2.5 kb region 5′ of the *exp-2* start codon for HNF4α-like consensus binding sites [37] and identified four putative elements (Figure 4B). To test whether NHR-69 directly binds any of these sites, we performed chromatin immunoprecipitation (ChIP) on mixed-stage *nhr-69p::nhr-69::gfp* animals using an anti-GFP antibody. NHR-69 was found to directly associate with at least two of these sites (Figure 4C). Intriguingly, NHR-69 binding was reduced, albeit not entirely lost, in *daf-8(m85)* mutants, suggesting that DAF-8 is required for optimal NHR-69 binding to the *exp-2* promoter (Figure 4C).

**Figure 5:**
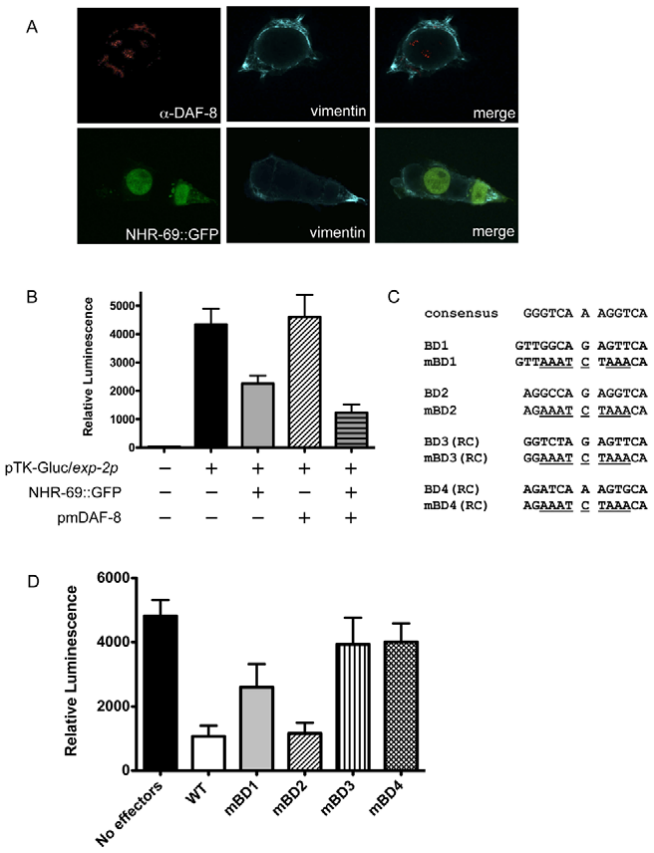
NHR-69 and DAF-8 suppress *exp-2* promoter activity in mammalian cells. (A) Plasmids expressing NHR-69::GFP and a phosphomimetic variant of DAF-8 (pmDAF-8) were transiently transfected into HEK293 cells and visualized by fluorescence imaging; both proteins show nuclear localization. (B) The bar graphs depict relative luciferase activity resulting from a transiently transfected *exp-2*-driven luciferase reporter in HEK293 cells. NHR-69, but not pmDAF-8 single transfection represses the *exp-2* promoter, and cotransfection of NHR-69 and pmDAF-8 causes an even stronger repression. (C) Details on the site-directed mutagenesis of HNF4α-like binding sites in the *exp-2* promoter. (D) The graph depicts the consequence of site directed mutagenesis of HNF4α-like binding sites in the *exp-2* promoter on luciferase activity. “No effectors” indicates transfection of the reporter alone, whereas “WT” indicates co-transfection of NHR-69 and pmDAF-8 with the wild-type *exp-2* promoter (similar to panel B). Mutating BD1, BD3, or BD4 attenuates the NHR-69 and pmDAF-8-mediated repression of *exp-2* activity. The result is an average from three individual trials.

To confirm that NHR-69 directly regulates the *exp-2* promoter we developed a mammalian reporter assay. We previously showed that the nuclear localization of DAF-8 requires the TGF-β Type 1 receptor DAF-1 [7], and others have reported that mutating two C-terminal serines (Ser465 and Ser467) to glutamates results in phosphomimetic, constitutively active human Smad2 [38]. Generating a phosphomimetic form of DAF-8 (pmDAF-8) by changing two orthologous C-terminal serines (S543 and S544) to glutamate followed by immunohistochemistry showed that pmDAF-8 localizes to the nucleus in transiently transfected HEK293 cells (Figure 5A; resembling the nuclear localization of a DAF-8::GFP fusion protein in *C. elegans* [7]). NHR-69::GFP was also localized to the nucleus when transiently transfected into HEK293 cells (Figure 5A). Importantly, NHR-69::GFP repressed the activity of a construct in which the wild-type *exp-2* promoter is fused to a luciferase reporter (Figure 5B). Moreover, this repression was enhanced by co-transfection of pmDAF-8 (Figure 5B), resembling the results obtained in *C. elegans*.

To test whether any of the NHR-69 binding sites identified in our ChIP analysis are functionally important, we performed a mutational analysis of the *exp-2* promoter (Figure 4B and Figure 5C). Using plasmids carrying *exp-2* promoter variants with mutated NHR-69 binding sites (BD1, BD2, BD3 or BD4) resulted in 46%, 76%, 18%, and 17% respective reductions of promoter activity upon NHR-69::GFP and pmDAF-8 co-transfection, whereas the wild-type *exp-2* promoter was repressed by approximately 80% (Figure 5D). These data suggest that BD1, BD3, and BD4 contribute to *exp-2* repression by NHR-69 whereas BD2 is likely dispensable.

### EXP-2 functionally contributes to insulin signaling downstream of NHR-69 and DAF-8

The above data indicate that *exp-2* is a direct target of *nhr-69* and *daf-8;* thus, we next wished to test whether *exp-2* was functionally required downstream of *daf-8* and *nhr-69*. EXP-2 is a potassium channel that regulates the action potential in pharyngeal muscle [36]. Worms carrying a heterozygous *exp-2* gain-of-function allele (*exp-2(sa26)*, encoding an EXP-2 protein with a C480Y point mutation) exhibit defective feeding behavior, hyperactive head movements, and egg-laying defects, whereas homozygous worms arrest terminally after hatching [36] [39]. Worms carrying null mutations in *exp-2*, such as the *exp-2(sa26ad1426)* allele carrying a T457I mutation, exhibit no defects in defecation or head movement, but have long-lasting pharyngeal muscle contractions [36]. Together, these mutants provide an opportunity to address the functional requirement of *exp-2* in insulin signaling.

**Figure 6:**
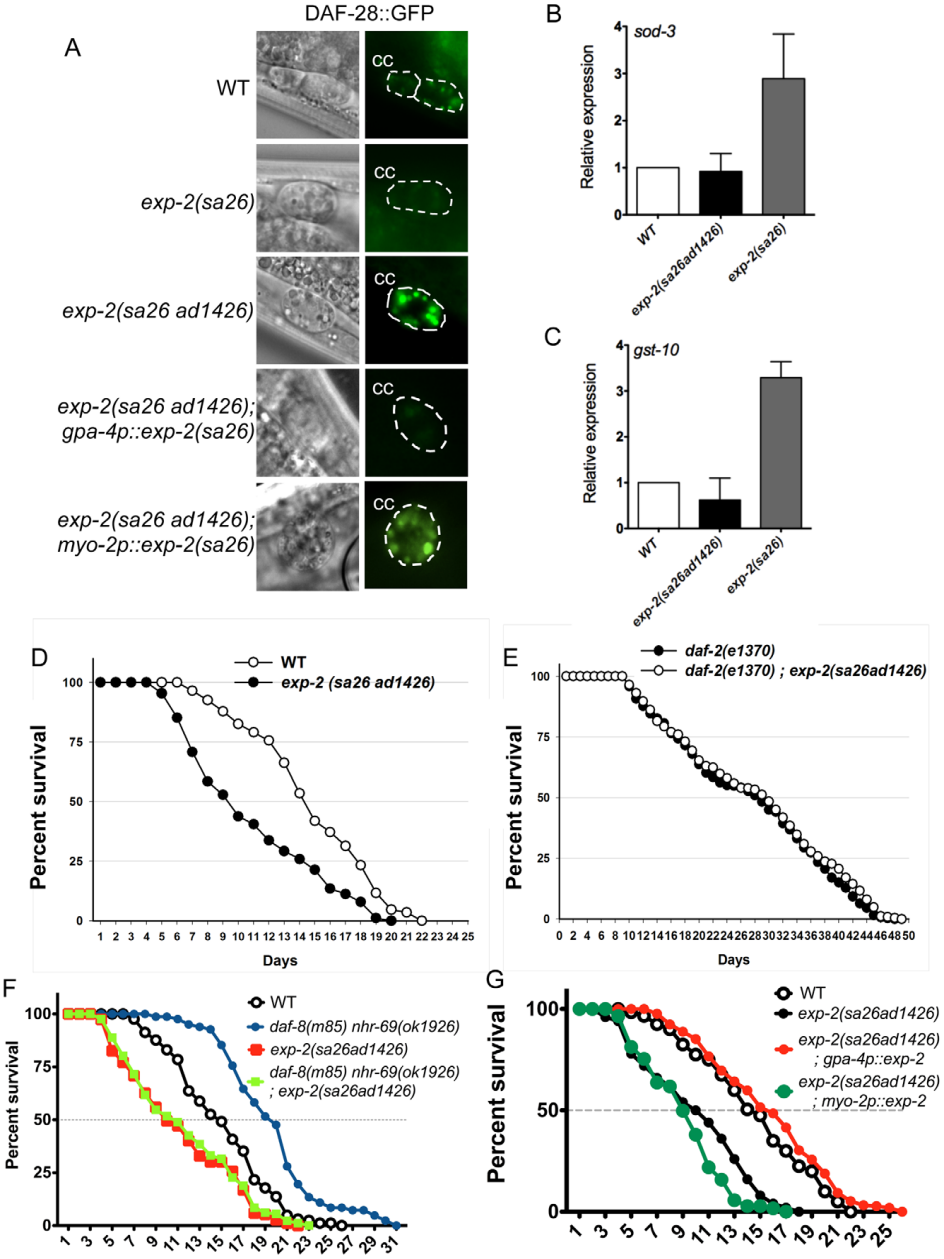
Insulin signaling is impaired in *exp-2* mutants. (A) The micrographs show DAF-28::GFP accumulation in coelomocytes (CC) in wild-type worms, in *exp-2* mutants, and in *exp-2* transgenic strains. *exp-2(sa26ad1426)* loss-of-function and *exp-2(sa26)* gain-of-function mutants show increased and decreased DAF-28::GFP intensity, respectively, indicating that EXP-2 represses DAF-28 secretion. ASI-neuron specific expression (driven by the *gpa-4* promoter) of the gain-of-function *exp-2(sa26)* allele in the *exp-2(sa26ad1426)* background reduces DAF-28::GFP accumulation, resulting in a level similar to that seen in the global *exp-2(sa26)* gain-of-function mutant; in contrast, pharyngeal-specific *exp-2(sa26)* expression (driven by the *myo-2* promoter) does not alter DAF-28::GFP accumulation in the *exp-2(sa26ad1426)* background. (B and C) The bar graphs depict average *sod-3* and *gst-10* mRNA levels in wild-type (white), *exp-2(sa26ad1426)* (black), and *exp-2(sa26)* (grey) worms, as determined by qPCR (normalized to *act-2*). Error bars indicate SEM from four biological replicates. (D) The graph depicts population survival curves for wild type worms (white) and *exp-2(sa26ad1426)* loss-of-function mutants (black; T = 25.5°C). (E) The graph shows population survival curves of *daf-2(e1370)* (black) and *daf-2(e1370); exp-2(sa26ad1426)* mutants (white; T = 25.5°C). (F) The graph depicts population survival curves of wild-type (black circle), *daf-8(m85) nhr-69(ok1926)* (blue circle), *exp-2(sa26ad1426)* (red square), and *daf-8(m85) nhr-69(ok19260; exp-2(sa26ad1426)* (green square) worms (T = 25.5°C). (G) The graph shows population survival curves of wild-type (white), *exp-2(sa26ad1426)* (black), *exp-2(sa26ad1426); gpa-4p::exp-2* (red) and *exp-2(sa26ad1426); myo-2p::exp-2* (green) (T = 25.5°C). All strains shown in this panel also harbor the *pRF4[rol-6(su1006)]* transgene.

To study the effect of *exp-2* loss, we generated a *daf-8(m85) nhr-69(ok1926) exp-2(sa26ad1426)* triple mutant and found that the life-span extension due to loss of *daf-8* and *nhr-69* was abolished by concomitant loss of *exp-2* (Figure 6F, Table S1). Taken together, our results suggest that NHR-69 and DAF-8 cooperate to directly regulate *exp-2*, and that *exp-2* is a critical downstream target of these two transcription factors.

### EXP-2 regulates insulin signaling through its actions in the ASI neurons

Because the *exp-2* loss-of-function mutation reduces the longevity of *daf-8(m85) nhr-69(ok1926)* double mutants, we next tested whether *exp-2* directly affects insulin signaling. First, we tested whether loss-of-function and gain-of-function *exp-2* mutants [36] affect the accumulation of the agonistic insulin-like peptide DAF-28 in coelomocytes. Using a translational DAF-28::GFP reporter [40], we found that gain-of-function *exp-2(sa26)* mutants accumulated less DAF-28::GFP in the coelomocytes than wild-type worms, whereas loss-of-function *exp-2(sa26ad1426)* mutants accumulated more (Figure 6A, Figure S5A).

Because NHR-69 and DAF-8 directly regulate *exp-2*, and because DAF-28, DAF-8 and NHR-69 are expressed in ASI neurons (Figure S2) [7] [32], we reasoned that EXP-2 should also modulate DAF-28::GFP secretion by acting in the ASI neurons. In support of this possibility, *exp-2* is strongly expressed in the amphid neurons, as well as in the pharyngeal muscle [36]. To address a putative tissue-specific role for EXP-2, we expressed the *exp-2(sa26)* mutant gene under the control of the ASI-specific *gpa-4* promoter [*gpa-4p::exp-2(sa26)*] [41] or the pharynx-specific *myo-2* promoter [*myo-2p::exp-2(sa26)*] [42] in *exp-2(sa26ad1426); daf-28::gfp* animals. We found that, when *exp-2(sa26)* was expressed selectively in ASI neurons, it caused DAF-28::GFP accumulation at a similar level as seen in *exp-2(sa26)* worms, whereas pharyngeal expression of *exp-2(sa26)* resulted in DAF-28::GFP accumulation comparable to that observed in *exp-2(sa26ad1426); daf-28::gfp* animals (Figure 6A, Figure S5A). Importantly, the mRNA level of *daf-28* was comparable in wild-type, *exp-2(sa26)*, *exp-2(sa26ad1426)*, *gpa-4p::exp-2(sa26)* and *myo-2p::exp-2(sa26)* worms, as determined by qPCR (Figure S5B). Thus, EXP-2 affects DAF-28 secretion, but not expression, selectively through its function in the ASI neurons.

If EXP-2 regulated DAF-28 secretion, it would be expected to affect downstream insulin target genes and phenotypes such as longevity. Indeed, the mRNA levels of *sod-3* and *gst-10* were upregulated in gain-of-function *exp-2(sa26)* mutants (Figure 6B, 6C), and we also observed increased nuclear localization of DAF-16::GFP in the *exp-2(sa26)* background (Figure S6), consistent with decreased insulin signaling [43]. Moreover, *exp-2(sa26ad1426)* loss-of-function mutants exhibited a significantly shortened life span compared to that of wild-type worms (mean life span: 9.4±0.6, p = 0.0116; we were unable to assess adult life span of *exp-2(sa26)* mutants due to lethality). In contrast, the life span of the *exp-2(sa26ad1426); daf-2(e1370)* double mutants was comparable to that of the *daf-2(e1370*) mutant itself (Figure 6E, p = 0.7606), suggesting that *exp-2* acts upstream of *daf-2* to regulate life span, consistent with its role in DAF-28 secretion.

Because *exp-2* acts selectively in ASI neurons to control DAF-28 secretion (Figure 6A), we next addressed whether life span regulation by *exp-2* also originates from the ASI neurons. Expression of wild-type *exp-2* specifically in the ASI neurons *(gpa-4p::exp-2)* rescued the short life span phenotype of *exp-2(sa26ad1426)* mutant worms (14.3 days, p = 0.0139), whereas pharyngeal-specific expression did not (8.7 days, p = 0.8326; Figure 6G). This suggests that *exp-2* plays an important role in ASI-neurons to regulate adult life span, possibly through its effect on DAF-28 secretion. Taken together, these data provide evidence that life span is inversely related to EXP-2 activity.

### Overexpression of NHR-69 in the ASI neurons confers a hypoglycemic phenotype

**Figure 7:**
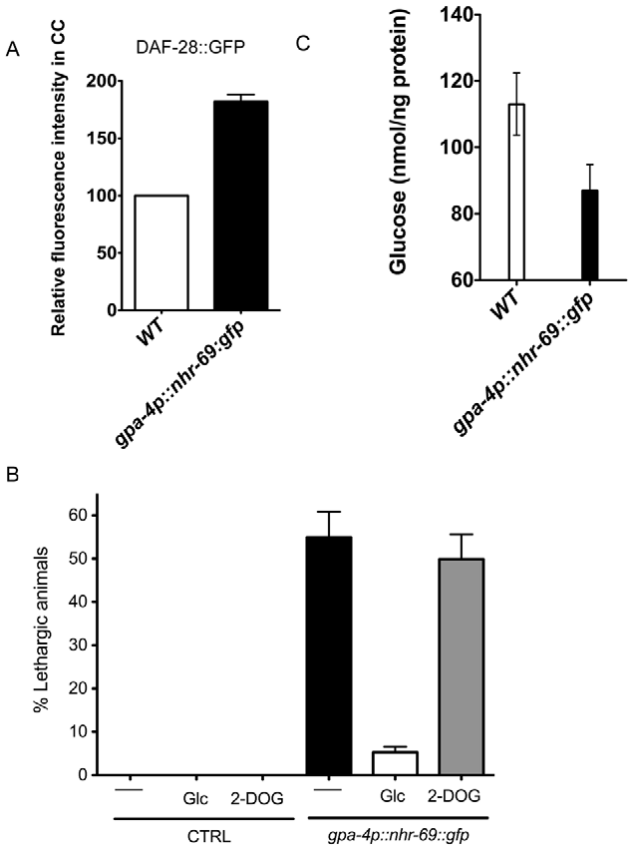
ASI-specific over-expression of NHR-69 confers hypoglycemia. (A) The bar graphs show the relative DAF-28::GFP level in coelomocytes (CC) in wild-type and *mIs40[gpa-4p::nhr-69::gfp]* day-1 old adult worms. Error bars indicate SEM. N = 62 in wild type, N = 77 in *mIs40[gpa-4p::nhr-69::gfp]* backgrounds (p<0.001). (B) Bars indicate the percentage of lethargic animals in the presence or absence of exogenous glucose (2 mM Glc) or 2-dexoyglucose (2 mM 2-DOG). Synchronous day-1 adults were video recorded for two minutes individually and worms that stopped and resumed moving more than four times were judged to be lethargic (N = 50). (C) Bars show endogenous glucose content in wild-type and *mIs40[gpa-4p::nhr-69::gfp]* worms. Synchronous day-1 adults were assayed in four independent biological replicates. Error bars indicate SEM.

As NHR-69::GFP is expressed in ASI neurons and DAF-28 is produced and secreted in these cells [32], we asked whether NHR-69 activity in the ASI neurons directly affects insulin signaling. First, we wished to ensure that an *nhr-69::gfp* translational fusion was biologically functional; thus, we expressed *nhr-69::gfp* from its own promoter (*mIs39[nhr-69p::nhr-69::gfp]*). Figure 3C shows that worms expressing this construct in the *daf-8(m85) nhr-69(ok1926)* mutant background exhibited a shortened life span compared to wild-type and a normal dauer recovery phenotype, suggesting that functional NHR-69 was overexpressed in these worms (Figure 3C and Table 2). Having assured that *nhr-69* rescue can work in principle, we generated two transgenic lines that expressed NHR-69::GFP under the control of the ASI-specific *gpa-4* promoter (*gpa-4p::nhr-69::gfp*) [41]. In these worms, we observed a higher accumulation of DAF-28::GFP in coelomocytes (compared to the wild-type background; Figure 7A). Moreover, the ASI specific expression of *nhr-69::gfp* resulted in a lethargic phenotype with slow, sluggish movement (Figure 7B) reminiscent of hypoglycemia in mammals [44]. Indeed, the endogenous glucose content was reduced in *gpa-4p::nhr-69::gfp* expressing animals compared to that of wild type (Figure 7C). In agreement with lower glucose levels in *gpa-4p::nhr-69::gfp* animals, the lethargic phenotype was almost completely rescued by exogenous glucose, but not by non-metabolizable 2-deoxyglucose, suggesting that glucose metabolism, not uptake is impaired in these animals. We conclude that NHR-69 overexpression in the ASI neurons results in hypoglycemia manifested as lethargic behavior, and that NHR-69 acts in the ASI neurons to regulate DAF-28 secretion.

## Discussion

The combinatorial interactions of Smad proteins with other DNA-binding transcription factors provide a critical selectivity required to regulate specific subsets of target genes. In this regard, identifying the transcriptional regulators that cooperate with Smad proteins will help delineate the mechanisms underlying signal integration. Here, we describe a molecular crosstalk in *C. elegans* whereby the R-Smad DAF-8 partners with NHR-69 to regulate insulin signaling. We find that NHR-69 and DAF-8 regulate the *exp-*2 potassium channel gene, which in turn modulates the secretion of the insulin-like peptide DAF-28. We further demonstrate that NHR-69 affects DAF-28 secretion and glucose homeostasis through its actions in the neuropeptide-secreting ASI neurons that functionally resemble the mammalian pancreas. These data not only implicate the HNF4α-like NHR-69 in insulin signaling, but also suggest a novel mechanism for the previously suggested link between IIS and TGF-β signaling. Intriguingly, Narasimhan *et al.* recently showed that the phosphatase PDP-1 modulates IIS by acting at the level of the Smad proteins DAF-8 and DAF-14 [13]. Taken together with our findings, this strengthens the notion that there is significantly more crosstalk between IIS and TGF-β signaling than previously appreciated.

### DAF-8 and NHR-69 cooperatively regulate insulin signaling

We set out to find novel transcriptional partners for DAF-8 and identified NHR-69 as a candidate DAF-8 interacting protein. *In vivo* co-immunoprecipitation and *in vitro* GST pull-down assays confirmed that NHR-69 and DAF-8 physically associate, whereas the Smads DAF-3 and DAF-14 did not bind to NHR-69, indicating that the interaction is specific for DAF-8. We also found evidence for a functional interaction between DAF-8 and NHR-69, demonstrating that *nhr-69* is involved in dauer formation: depletion of *nhr-69* by RNAi enhanced dauer formation in sensitized genetic backgrounds, including *sdf-9* and several TGF-β Daf-c mutants. Furthermore, resumption of normal larval development of *daf-1*, *daf-7*, *daf-8* and *daf-14* dauer larvae after temperature downshift was delayed approximately two-fold in the *nhr-69* mutant background. Because *daf-2* signaling is required for temperature-dependent dauer recovery [27], this result suggests that insulin signaling was impaired in *nhr-69* mutants.

In line with this notion, we observed an extended life span and upregulation of the insulin-regulated genes *sod-3* and *gst-10* in the *daf-8 nhr-69* double mutant. We note that the observed longevity likely reflects altered expression of genes other than just *sod-3* and *gst-10*, akin to the observations made in long-lived *daf-2* mutants, wherein numerous genes are thought to contribute to longevity [9]. Lastly, we observed a reduced accumulation of ANF::GFP in the coelomocytes of *daf-8 nhr-69* double mutants, indicating that neuropeptide secretion is impaired in *daf-8 nhr-69* double mutants, representing the likely cause for impaired insulin signaling.

The relationship between TGF-β signaling and IIS may be conserved in evolution. Members of the TGF-β superfamily affect the development and physiology of mammalian pancreatic β-cells, although the results are somewhat conflicting. In one study, Smad3 repressed the insulin gene promoter and Smad3 knockdown enhanced glucose stimulated insulin secretion (GSIS) [45], suggesting a repressive role for TGF-β signaling. However, in isolated rat fetus pancreatic β-cells, TGF-β1 stimulates long-term (48 hours) insulin secretion [46], perhaps through transcriptional changes. Similarly, TGF-β1 and Activin A, another TGF-β family member, stimulate short-term (five minutes) insulin secretion in mouse insulinoma cells by increasing cytoplasmic Ca^2+^ concentration [47] [48]. In addition, conditional expression of the inhibitory Smad7 in β-cells resulted in reduced pancreatic insulin levels and in hypoinsulinemia. Lastly, expression of the TGF-β related bone morphogenetic protein 4 (BMP4) and its receptor BMPR1A regulate glucose metabolic genes to enhance GSIS and promote insulin signaling [49]. Our results are more consistent with the latter reports as the *daf-8 nhr-69* double mutant exhibited decreased overall insulin signaling.

### NHR-69 and DAF-8 control *exp-2* expression to regulate DAF-28 secretion

Mechanistically, several lines of evidence suggest that NHR-69 and DAF-8 influence DAF-28 secretion by directly regulating the expression of *exp-2*, which encodes a voltage-activated (Kv-type) potassium channel. Using ChIP, we found that NHR-69 associated with at least two elements in the *exp-2* promoter region that resemble consensus HNF4α binding sites; moreover, this binding was reduced in *daf-8* mutants. *daf-8 nhr-69* double mutants synergistically induce *exp-2* expression, suggesting that *exp-2* is repressed by these two factors. Interestingly, using expression profiling, Shaw et al. previously found *exp-2* to be induced in several TGF-β Daf-c mutants, including *daf-8* mutants [9], although they did not report the extent of the upregulation (we observed a mild induction in *daf-8* mutants that was not statistically significant; see Figure 4A). Lastly, we used a heterologous cell culture system to confirm that NHR-69 and DAF-8 collaboratively repress *exp-2*, with three distinct NHR-69 binding sites being required for full repression.

Together, our results imply that NHR-69 and DAF-8 assemble a complex on the *exp-2* promoter that represses *exp-2* transcription. Due to the divergent nature of the DNA-binding domain of DAF-8 (only 28% similar to the MH1 domain of human Smad1, and 30% similar to the MH1 domain of *Drosophila melanogaster* Mad1; Park & Taubert, unpublished), it is difficult to predict whether DAF-8 would bind to the consensus Smad-binding-element (SBE) [50]. Further experiments are required to assess whether DAF-8 directly binds the *exp-2* promoter or whether it is tethered to this region through an interaction with NHR-69.

Our data further indicate that *exp-2* is critical for insulin signaling downstream of DAF-8 and NHR-69 (but upstream of DAF-2). Firstly, the *exp-2(sa26ad1426)* mutation, which shortens the life span of wild-type worms, did not shorten the life span of *daf-2* mutants. Secondly, *exp-2(sa26ad1426)* and *exp-2(sa26)* mutants altered DAF-28 secretion. Thirdly, *exp-2(sa26)* mutants exhibited increased nuclear localization of DAF-16/FoxO, a typical consequence of reduced DAF-2 activity. Lastly, the *exp-2(sa26)* mutant showed increased expression of two downstream DAF-2 target genes. Thus, we find evidence for a novel regulatory relationship between insulin secretion and TGF-β signaling. As *nhr-69* transcription is itself induced by insulin signaling, a positive regulatory loop to promote larval development and prevent dauer formation is initiated, but only when environmental stimuli activate both TGF-β and insulin signaling.

EXP-2 is a member of the six-transmembrane, voltage-activated potassium channel family, which also controls muscle contraction in the *C. elegans* pharynx. EXP-2 produces an outward current at the plasma membrane, which terminates the action potential by repolarizing the membrane [36]. As EXP-2 exhibits homology to multiple mammalian Kv-type channels it is difficult to provide a direct parallel to the functions of one mammalian channel. However, several Kv-type channels are known to affect insulin signaling and/or glucose balance [51]. Most prominently, pharmacological inhibition or genetic disruption of Kv2.1 causes increased glucose-stimulated insulin secretion [52] [53]. In the future, it will be interesting to test whether NHR action and TGF-β signaling coregulate these mammalian channels.

### Neuronal NHR-69 overexpression affects glucose homeostasis and DAF-28 secretion

The expression of *nhr-69* overlaps with that of *daf-8* in ASI neurons and in the intestine [7], suggesting that the molecular crosstalk between DAF-8 and NHR-69 may occur in these two tissues. Cell ablation analyses demonstrated that the ASI neurons inhibit dauer formation [54], and are required for the expression of the TGF-β ligand DAF-7 and of the insulin-like peptide DAF-28, both of which promote reproductive growth [55] [32]. Because of the latter property, the ASI neurons have been proposed to functionally resemble the insulin-producing pancreatic β-cells [40]. To test whether NHR-69 was functionally important in the ASI neurons, we generated a strain that overexpresses *nhr-69* specifically in these neurons. These worms exhibited greater accumulation of DAF-28::GFP, a lethargic phenotype, and reduced glucose levels, reminiscent of a hyperinsulinemic/hypoglycemic phenotype [44]. NHR-69 may achieve these effects by regulating other neuropeptides in addition to DAF-28; in any case our results suggest that the regulation of NHR-69 levels and activity in the ASI neurons is critical to achieve glucose homeostasis in *C. elegans*. They also support the notion that ASI neurons exhibit characteristics of mammalian pancreatic β-cells [40]. We note, however, that NHR-69 and DAF-28 are also co-expressed in the intestine, a tissue that plays a key role in metabolic regulation in *C. elegans* and may also perform pancreas related functions.

**Figure 8:**
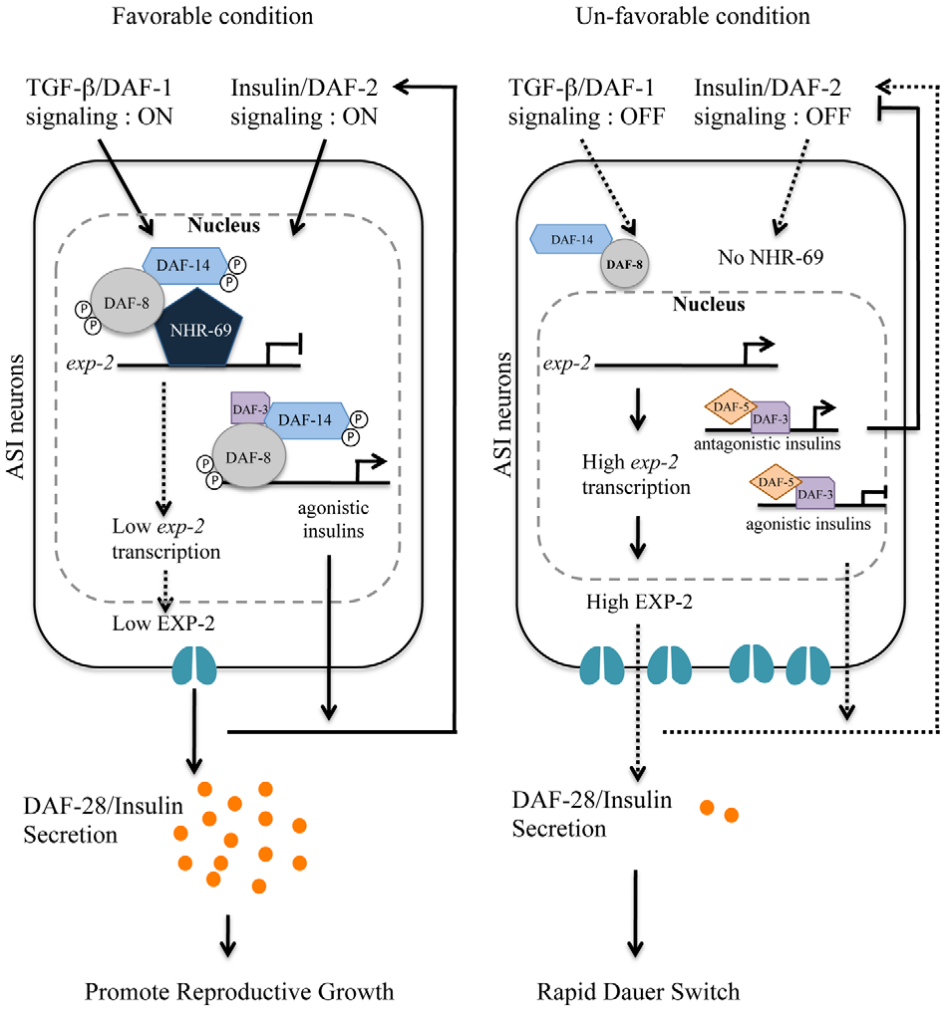
Working model for DAF-8 and NHR-69 modulation of insulin secretion in *C. elegans*. Based on our data, we propose that DAF-2 signaling positively regulates transcription of *nhr-69*, whereas the TGF-β receptor is known to phosphorylate DAF-8 to activate its function [7]. NHR-69 and DAF-8 cooperatively repress the transcription of the *exp-2* Kv channel gene in ASI neurons, which in turn causes sustained secretion of the insulin-like peptide DAF-28, hence activating a feed-forward loop on DAF-2 in favorable conditions. TGF-β and Insulin/IGF-1 signaling crosstalk at the *exp-2* promoter through the DAF-8 and NHR-69 transcription factors. Upregulation of *exp-2* by the reduction of DAF-8 and NHR-69 activity would attenuate DAF-28 secretion, providing a fast decision for the dauer formation.

Our overall model (Figure 8) integrates the observations described above: in favorable conditions, DAF-8 and NHR-69 cooperate to repress *exp-2* transcription in the ASI neurons, thus causing sustained DAF-28 secretion, which in turn activates IIS and promotes reproductive growth. This mechanism would act in parallel to the one proposed by Narasimhan et al., whereby DAF-8 promotes IIS and reproductive growth by directly inducing the transcription of agonistic insulin peptide genes such as *ins-4* [13]. In harsh conditions, *exp-2* transcription is de-repressed as a consequence of reduced DAF-8 phosphorylation and lower *nhr-69* expression; increased EXP-2 activity would presumably lead to a rapid reduction in the overall levels of agonistic insulins, thus providing a rapid signal for dauer formation (Figure 8).

### NHR-69 functionally resembles pancreatic HNF4α

The *C. elegans* genome encodes 284 NHRs, most of which derive from an HNF4α-like ancestor. The human *HNF4A* gene encoding HNF4α is mutated in a subtype of MODY (Maturity Onset Diabetes of the Young) and HNF4α is a central regulator of hepatic and pancreatic gene transcription [37]. MODY patients fail to properly secrete insulin in response to glucose challenge [56] and β-cell-specific knockout of HNF4α in mice results in reduced GSIS [57] [58]. Intriguingly, akin to our discovery of physical and functional DAF-8:NHR-69 interaction, HNF4α and Smad3 interact physically and functionally in transfected human cells [59], although the physiological impact of this interaction remains unclear. Our study suggests that such HNF4α:Smad complexes may affect glucose homeostasis by regulating potassium channel genes in pancreatic β-cells.

The functions of most *C. elegans* HNF4α-related NHRs remain unknown and no *C. elegans* NHR has yet been implicated in glucose and/or insulin metabolism. We provide several lines of evidence that NHR-69 performs these particular functions, somewhat resembling pancreatic HNF4α. Most compellingly, we observe hypoglycemia in animals that overexpress NHR-69 specifically in ASI neurons [32] [40]. However, we note that, compared to HNF4α, the defects in *nhr-69* single mutants (and in *nhr-69* heterozygotes) are relatively mild indicating that possibly other NHRs such as the closely related NHR-64 act redundantly or can compensate for the loss of *nhr-69*. Given the vast number of HNF4α-related NHRs in *C. elegans*, other NHRs may also control dauer formation, especially in a sensitized genetic background. As many *C. elegans* NHRs form heterodimers [60], we speculate that NHR-69 may form heterodimers to implement its regulatory roles.

Human HNF4α targets hundreds of genes in the liver and pancreas to modulate metabolic processes such as lipid biosynthesis, glucose homeostasis and insulin secretion [37]. However, the molecular and physiological functions of individual HNF4α target genes and their combinatorial regulation remains poorly understood. Identifying the functions and the target genes of *C. elegans* HNF4α-like NHRs can provide insights into the mechanism of related mammalian NHRs. We recently proposed that the functions collectively executed by mammalian HNF4α might be distributed amongst individual *C. elegans* HNF4α orthologs [17]. For example, NHR-31 regulates the expression of a vacuolar ATPase in the excretory cell in *C. elegans* [61], possibly hinting at a similar regulatory role of mammalian HNF4α in kidney physiology. Our results suggest that NHR-69 may be a partial functional ortholog of pancreatic HNF4α. Thus, NHR-69 may provide a useful tool to dissect pancreatic HNF4α function, to define *in vivo* regulators of HNF4α and to delimit additional downstream targets that participate in the regulation of glucose homeostasis.

## Materials and Methods

### Nematode strains

*C. elegans* strains were cultured according to standard techniques [62] unless otherwise noted. The *E. coli* strain OP50 was used as a food source in all assays. Nematode strains and alleles used in this study are LG I: *daf-8(sa343)*, *daf-8(m85), nhr-69(ok1926) daf-16(mgDf47)*; LG III: *daf-7(e1372), daf-2(e1370)*; LG IV: *daf-1(m40), eri-1(mg366), daf-14(m77)*; LG V: *exp-2(sa26)/eT1[let-?(n886)], exp-2(ad26ad1426), sdf-9(m708). mIs1[rol-6(su1006)], mIs39[nhr-69p::nhr-69::gfp, rol-6(su1006)], mIs40[gpa-4p::nhr-69::gfp, rol-6(su1006)], mIs4[rol-6(su1006)], mIs27[daf-8p::daf-8::gfp, rol-6(su1006)], nhr-69(ok1926);daf-1(m40), nhr-69(ok1926); daf-7(e1372), daf-8(m85) nhr-69(ok1926), daf-8(m85) nhr-69(ok1926); mIs39, nhr-69(ok1926);daf-14(m77), daf-7(e1372); mIs39, daf-2(e1370);mIs39, daf-8(m85) nhr-69(ok1926); oxIs206[aex-3p::ANF::GFP], daf-8(m85) nhr-69(ok1926); arIs37[myo-3p::ssGFP], exp-2(sa26); svIs69[daf-28p::daf-28::gfp], exp-2(sa26ad1426); svIs69[daf-28p:daf-28::gfp], daf-2(e1370); exp-2(sa26ad1426), daf-8(m85) nhr-69(ok1926); exp-2(sa26ad1426) exp-2(sa26); zIs356[daf-16p::daf-16::gfp], mIs40; svIs69.*

In the *nhr-69(ok1926)* mutant, 1329 bp are deleted, including the 5′ upstream regulatory sequences and the first four exons (www.wormbase.org). The nonsense mutation *daf-8(m85)* is predicted to truncate DAF-8 in the conserved MH2 domain. It is the strongest *daf-8* allele, forming virtually 100% dauer larvae at 25.5°C [7].

### Transgene construction and transformation

A genomic fragment containing the entire coding region of *nhr-69* plus 3 kb upstream of the ATG was amplified. The *gfp* gene was amplified from pPD95.75 (kindly provided by A. Fire) and used for PCR fusion (see [63]) to generate *nhr-69p::nhr-69::gfp*, a translational fusion of *gfp* at the end of *nhr-69* exon 9. The *gpa-4p::nhr-69::gfp* fusion construct was generated by the same method, with a 2.8 kb 5′ *gpa-4* promoter region [41] being used. The fusions were introduced by microinjection into the adult germ line (45 ng/µl) along with the injection marker *pRF4* [*rol-6* (*su1006*)] (9 ng/µl).

### Life span analysis and measurement of glucose content

Eggs were prepared by alkaline hypochlorite treatment and synchronized by hatching in M9 buffer. After growth on OP50 at 15°C, animals at mid-L4 stage were transferred to NG agar plates containing 40 µM of 5-fluoro-2′-deoxyuridine (FUdR) to prevent progeny production [64]. Life span experiments were performed at 25°C as previously described [65]. We averaged mean life spans from three independent biological replicates. Representative survival curves are shown. We used one-way analysis of variance (ANOVA) for statistical analysis of mean life spans ANOVA [66].

We measured total worm glucose levels with a Glucose Assay Kit (Biovision K606–100). Worms grown on NG agar plates were collected and washed five times in M9 buffer. The worm pellet was resuspended in 500 µl of glucose assay buffer, frozen at −80°C, thawed on ice, and then sonicated (Branson Sonifier 450) five times (output level 3, time 10 seconds) to obtain extracts. After centrifugation at 13,000 rpm at 4°C, the supernatant was used for the glucose assay according to the manufacturer's instructions. We normalized glucose content by protein content in each sample. Statistics were performed using GraphPad Prism 5.

### Lethargy assays

Worms were grown synchronously at 20°C from L1 to L4 stage, and then transferred to 25°C. Individual day-1 old adults were video recorded for two minutes, and were judged to be lethargic if they stopped and resumed moving more than four times (N = 50). The plates were tapped manually ten seconds prior to video recording to stimulate movement. For assaying suppression of lethargy by glucose, worms were grown as above and transferred to NG-agar plates supplemented with either 2 mM of D-(+)-Glucose (Sigma, G8270) or 2 mM of 2-deoxy-D-glucose (Sigma D8375).

### RNAi and dauer formation and recovery assays

Percent dauer formation was scored visually at 250× magnification for each genotype grown for two days from hatching at 20°C, 22.5°C, and 25.5°C. To assay dauer recovery, the dauer larvae were induced by growth at 25.5°C for each genotype. 50 dauer larvae were put on NG agar plates seeded with OP50 and incubated at 15°C. Dauer recovery was scored visually monitoring pharyngeal pumping and body size 24 and 48 hrs after transfer. RNAi feeding was performed using clones from the Ahringer library [67] on 1 mM Isopropyl β-D-1-thiogalactopyranoside (IPTG) as described [68].

### *In vitro* GST pull down, *in vivo* co-immunoprecipitation, and immunoblots

For *in vitro* GST pull down assays, full-length cDNAs encoding NHR-69 or Smad proteins were subcloned into pGEX-4T-1 (GE Healthcare Sciences) and pBluescript SKII vectors, respectively. The GST pull-down assay and *in vivo* co-IP were performed as previously described [7]. Immunoblots were probed with anti-GFP antibody (1∶2,000, Abcam ab6556) or anti-DAF-8 antibody (1∶1,000; a custom rabbit polyclonal anti-DAF-8 antibody raised against the peptide CQSNRQDDEEPGYYR, generated by GenScript). Total lysates were obtained by sonication (Branson, Sonifier 450). The anti-DAF-8 antibody specifically detected endogenous DAF-8 as well as the exogenous DAF-8::GFP fusion protein, but did not detect DAF-8 in the *daf-8(sa343)* mutant, in which the epitope is deleted (Figure S1).

### Chromatin immunoprecipitation (ChIP) and quantitative PCR

For ChIP, mixed-stage populations were subjected to a 1.5% formaldehyde solution for 30 minutes at room temperature to cross-link DNA and proteins [69]. We performed immunoprecipitation by incubating the lysate with either anti-GFP antibody (1∶250, Abcam) or IgG at 4°C for 16–18 hours. The precipitates were washed, the crosslinks reversed, and the DNA eluted. 5 µl of each eluate were used for quantitative PCR (qPCR).

For qPCR, 1 µg total RNA from each sample was used for reverse transcription with Superscript II reverse transcriptase (Invitrogen) and oligo dT priming. The resulting first-strand cDNA was analyzed by qPCR on an Applied Biosystems 7500 with BioRad iTaq SYBR green supermix using ROX as a normalization dye. The primers used for qPCR are listed in Table S2.

### Antibodies, immunohistochemistry, and promoter analysis in HEK293 cells

The phosphomimetic pmDAF-8 construct was amplified by PCR with primers dh_295_L and dh_296_R (Table S2). The resulting cDNA was cloned into the pcDNA3.1 (+) Neo vector using BamHI and EcoRI sites. The cDNA for *nhr-69::gfp* was amplified from *mIs39* animals by using dh_312_L and dh_313_R (Table S2). The resulting PCR product was cloned in the pcDNA3.1 (+) Hygro vector using NheI and ApaI sites. The wild-type 2.5 kb *exp-2* promoter was PCR amplified using dh_299_L/dh_300_R (Table S2) and subcloned in pTK-GLuc vector (New England BioLabs N8084) using KpnI and BamHI sites to generate pTK-Gluc/*exp-2*. The mutant *exp-2* promoter constructs were generated using a site directed mutagenesis kit (Invitrogen) with pTK-Gluc/*exp-2* as a template. The constructs were delivered to HEK293 cells by transient transfection using Lipofectamine (Invitrogen) according to the manufacturer's manual. The luciferase assays were performed using a BioLux Gaussia Luciferase Flex Assay Kit (New England BioLabs E3308L), and luminescence was measured in a Tecan M200 plate reader.

For immunofluorescent detection of labeled proteins, cells were fixed in 4% paraformaldehyde for 10 minutes, washed in PBS twice for five minutes per wash, permeabilized in 0.1% Triton-X-100 for 30 minutes, washed in PBS, and blocked in 4% Normal Goat Serum for 45 minutes, incubated with primary antibodies overnight in PBS/1% Normal Goat Serum. The following morning, the samples were washed twice with PBS and incubated with secondary antibodies for 1 hour, then washed 3×5 minutes in PBS. The coverslips were mounted using Vectashield (Vector Laboratory). The primary antibodies were: anti-DAF-8 (1/500) custom rabbit polyclonal antibody (Genscript), anti-Vimentin (1/200 Chicken IgY, Millipore #AB5733). The secondary antibodies were Alexa 546-coupled anti-rabbit IgG H+L, highly cross-adsorbed (1/200, Invitrogen/Molecular Probes #A11035), and donkey anti-chicken Cy5 (1/200, Millipore #AP194S).

### Microscopy

We used a Zeiss Axioscope equipped with a QImaging camera (RETIGA 2000R) for differential interference contrast (DIC) and fluorescence microscopy. ImageJ software was used to estimate the intensity of the GFP signal in coelomocytes. For DiI staining, cultures were synchronized by hatching purified eggs into M9 buffer, grown on NG agar plates until the L2 stage, washed in M9 buffer, and DiI stained, as described [70]. Fluorescent signals generated by immunostaining were visualized and captured using an Olympus FLUOVIEW FV10i confocal laser-scanning microscope system. Images from different color channels were colorized and merged using ImageJ software.

## Supporting Information

Figure S1 Anti-DAF-8 antibody specifically detects endogenous DAF-8. Mixed-stage worms were washed three times with M9 buffer and SDS sample buffer was added to the worm pellet. Total protein extracts were obtained by boiling. 40 µg of total protein was loaded into each well. The blot was probed with anti-DAF-8 antibody (1∶1,000).
(TIF)

Figure S2 *nhr-69p::nhr-69::gfp* and *daf-8p::daf-8::gfp* are expressed in the ASI neurons. Dorsal view of expression in ASI neuron (arrow in lower panel). The same animals were stained with DiI for ciliated neurons in the head (arrows in middle panel).
(TIF)

Figure S3 RNAi against *nhr-69* enhances dauer formation. The bar graphs show the percentage of wild type or *eri-1(mg366); sdf-9(m708)* worms that formed dauer larvae when grown on HT115 bacteria harboring control RNAi plasmid (No), or *gfp*, *akt-1*, or *nhr-69* RNAi plasmids. Worms were initially grown on OP50 at 15°C, transferred to lay eggs on plates with bacteria harboring the indicated RNAi construct, and then removed. The F1 progeny were grown to the L4 stage at 15°C; then, three F1 L4 larvae were transferred to fresh RNAi plates at 25°C, and removed as young adults after they had laid 30–40 eggs. Dauer formation in these F2 worms was then scored visually. *akt-1* RNAi was used as the positive control because we previously found that it promotes dauer formation in the *eri-1; sdf-9* background.
(TIF)

Figure S4 Accumulation of neuropeptide-GFP fusion proteins in coelomocytes (CC) and quantification of ANF::GFP expression. (A) Quantification of ANF::GFP in coelomocytes of wild type and *daf-8(m85) nhr-69(ok1926)* worms (black, p<0.001) indicates that the mutant strain is deficient in neuropeptide secretion. The *daf-8* (grey) and *nhr-69* single mutants (hatched) showed 10.2% and 15.4% reduction in the GFP intensity, respectively. (B) Quantification of coelomocyte ssGFP in wild type and *daf-8(m85) nhr-69(ok1926)* worms (p>0.05) shows that the difference seen in (A) is not due to a deficiency in coelomocyte uptake. The intensity in either *daf-8* (grey) or *nhr-69* (hatched) single mutant was comparable to that of wild type (p>0.05). (C) qPCR for *gfp* mRNA from animals expressing *aex-3p::ANF::GFP* in wild type and *daf-8 nhr-69*, *daf-8* and *nhr-69* (p>0.05) shows that the expression of intestinal peptides is similar in all strains. Bars indicate SEM for four independent experiments.
(TIF)

Figure S5 Coelomocyte DAF-28::GFP accumulation and *daf-28* expression in *exp-2* mutants. (A) Quantification of coelomocyte DAF-28::GFP in wild type worms, loss-of-function *exp-2(sa26ad1426)* and gain-of-function *exp-2(sa26)* mutants (p<0.001), and worms expressing *exp-2(sa26)* in ASI-specific (red bar, p>0.05 compared to *exp-2(sa26)* mutant) or pharyngeal-specific fashion in the *exp-2(sa26ad1426)* mutant background (green bar, p = 0.7966 compared to *exp-2(sa26ad1426)* mutant). (B) qPCR quantification of *daf-28* mRNA levels in wild-type, loss-of-function *exp-2(sa26ad1426)*, and gain-of-function *exp-2(sa26)* worms (p>0.05). Red and green bars represent mRNA levels in worms expressing *exp-2* in ASI-specific or pharyngeal-specific fashion, respectively. Error bars indicate SEM.
(TIF)

Figure S6 DAF-16::GFP exhibits nuclear localization in the gain-of-function *exp-2(sa26)* mutant.
(TIF)

Table S1 Life span phenotype described in the text.
(DOC)

Table S2 PCR Primers described in the text.
(DOC)
