# Supplementary figures and images for: Repression of a Potassium Channel by Nuclear Hormone Receptor and TGF-β Signaling Modulates Insulin Signaling in Caenorhabditis elegans
Source: PLoS Genet. 2012 Feb 16;8(2):e1002519. doi: 10.1371/journal.pgen.1002519 (PMC3280960; doi:10.1371/journal.pgen.1002519)

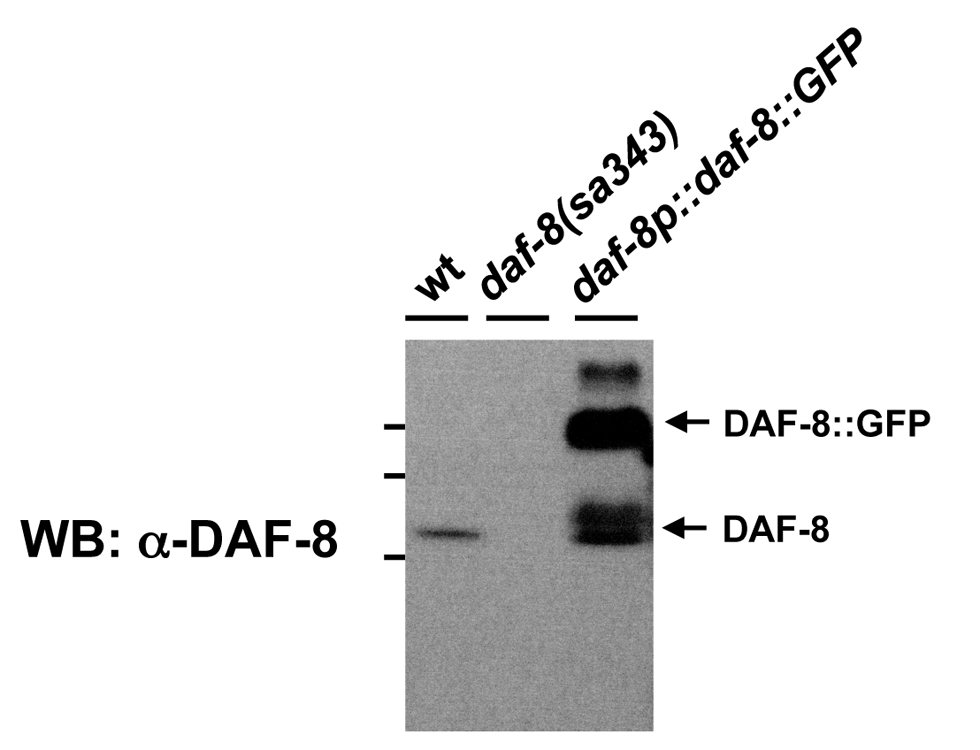

Supplement: Figure S1 — Anti-DAF-8 antibody specifically detects endogenous DAF-8. Mixed-stage worms were washed three times with M9 buffer and SDS sample buffer was added to the worm pellet. Total protein extracts were obtained by boiling. 40 µg of total protein was loaded into each well. The blot was probed with anti-DAF-8 antibody (1∶1,000). (TIF) [file pgen.1002519.s001.tif]

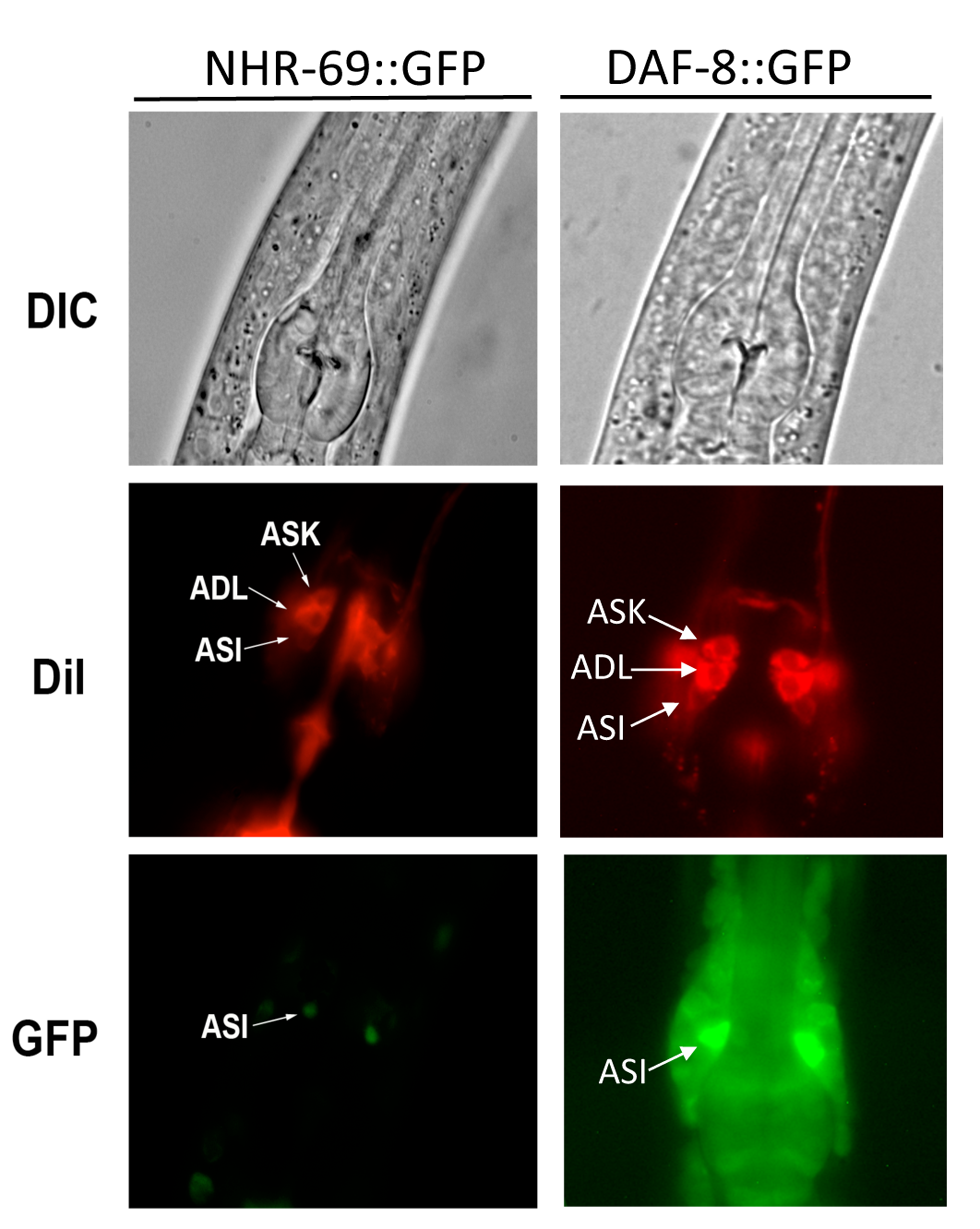

Supplement: Figure S2 — nhr-69p::nhr-69::gfp and daf-8p::daf-8::gfp are expressed in the ASI neurons. Dorsal view of expression in ASI neuron (arrow in lower panel). The same animals were stained with DiI for ciliated neurons in the head (arrows in middle panel). (TIF) [file pgen.1002519.s002.tif]

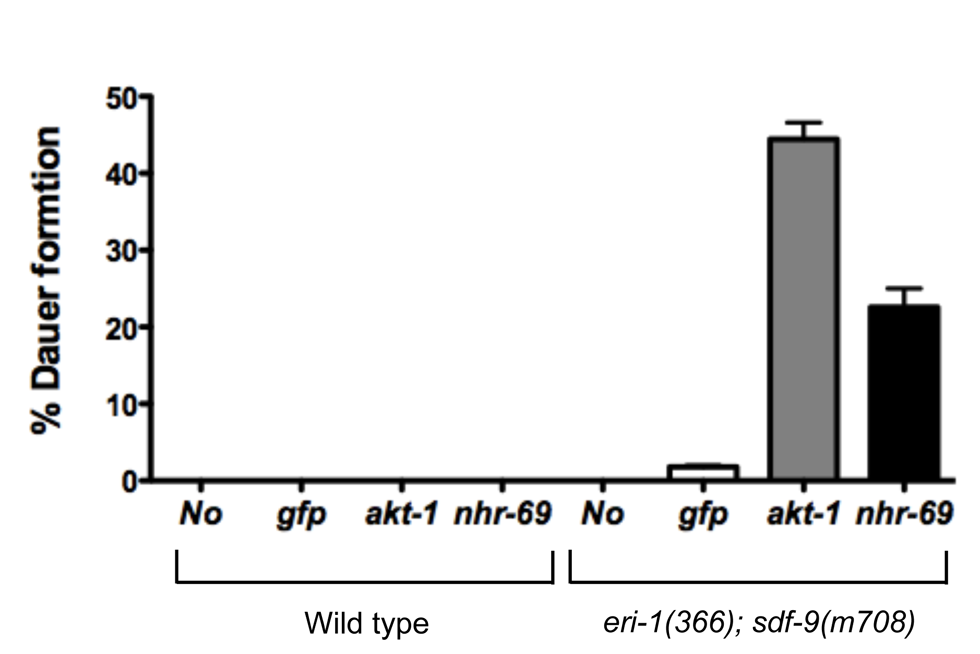

Supplement: Figure S3 — RNAi against nhr-69 enhances dauer formation. The bar graphs show the percentage of wild type or eri-1(mg366); sdf-9(m708) worms that formed dauer larvae when grown on HT115 bacteria harboring control RNAi plasmid (No), or gfp, akt-1, or nhr-69 RNAi plasmids. Worms were initially grown on OP50 at 15°C, transferred to lay eggs on plates with bacteria harboring the indicated RNAi construct, and then removed. The F1 progeny were grown to the L4 stage at 15°C; then, three F1 L4 larvae were transferred to fresh RNAi plates at 25°C, and removed as young adults after they had laid 30–40 eggs. Dauer formation in these F2 worms was then scored visually. akt-1 RNAi was used as the positive control because we previously found that it promotes dauer formation in the eri-1; sdf-9 background. (TIF) [file pgen.1002519.s003.tif]

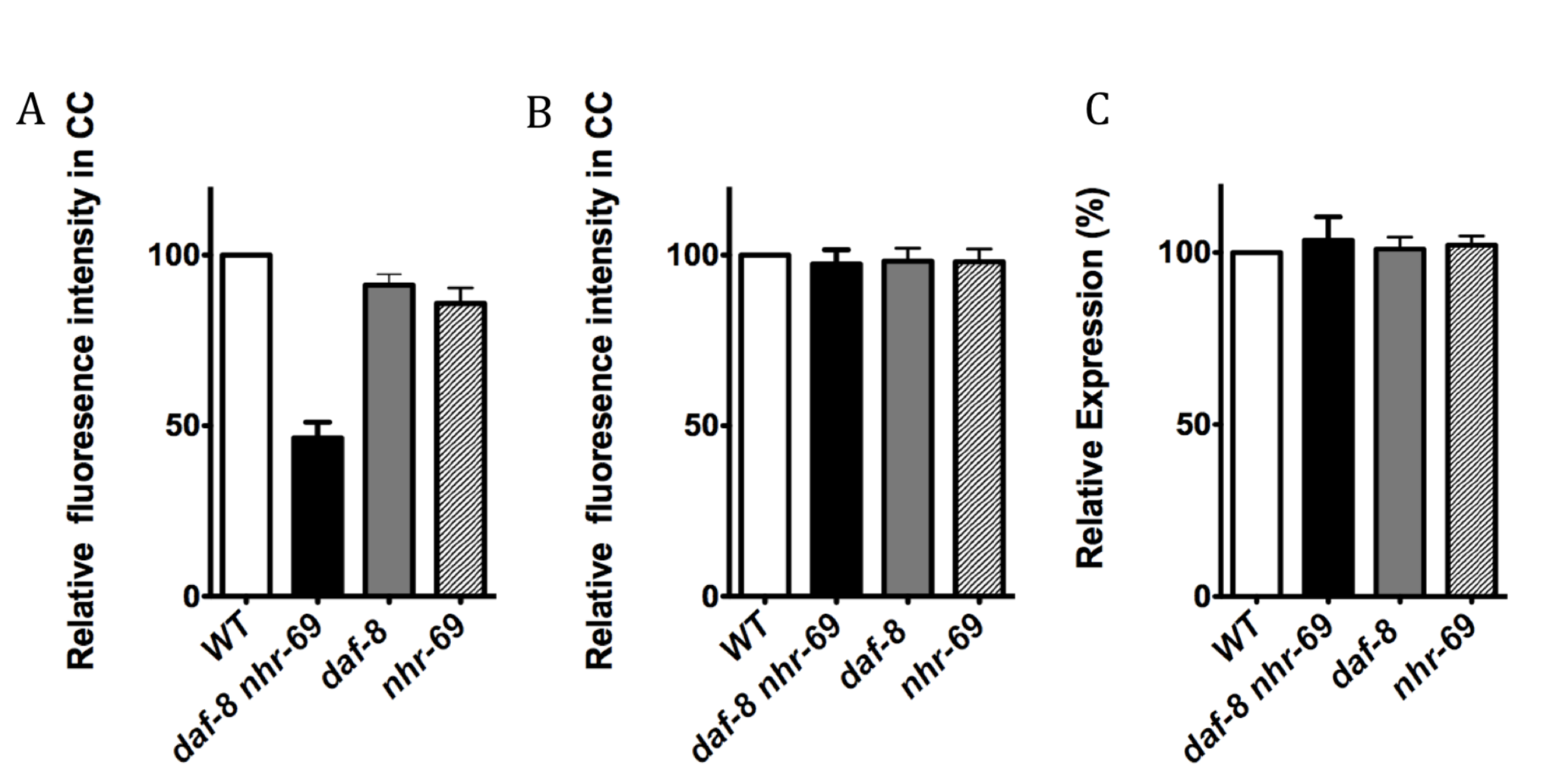

Supplement: Figure S4 — Accumulation of neuropeptide-GFP fusion proteins in coelomocytes (CC) and quantification of ANF::GFP expression. (A) Quantification of ANF::GFP in coelomocytes of wild type and daf-8(m85) nhr-69(ok1926) worms (black, p<0.001) indicates that the mutant strain is deficient in neuropeptide secretion. The daf-8 (grey) and nhr-69 single mutants (hatched) showed 10.2% and 15.4% reduction in the GFP intensity, respectively. (B) Quantification of coelomocyte ssGFP in wild type and daf-8(m85) nhr-69(ok1926) worms (p>0.05) shows that the difference seen in (A) is not due to a deficiency in coelomocyte uptake. The intensity in either daf-8 (grey) or nhr-69 (hatched) single mutant was comparable to that of wild type (p>0.05). (C) qPCR for gfp mRNA from animals expressing aex-3p::ANF::GFP in wild type and daf-8 nhr-69, daf-8 and nhr-69 (p>0.05) shows that the expression of intestinal peptides is similar in all strains. Bars indicate SEM for four independent experiments. (TIF) [file pgen.1002519.s004.tif]

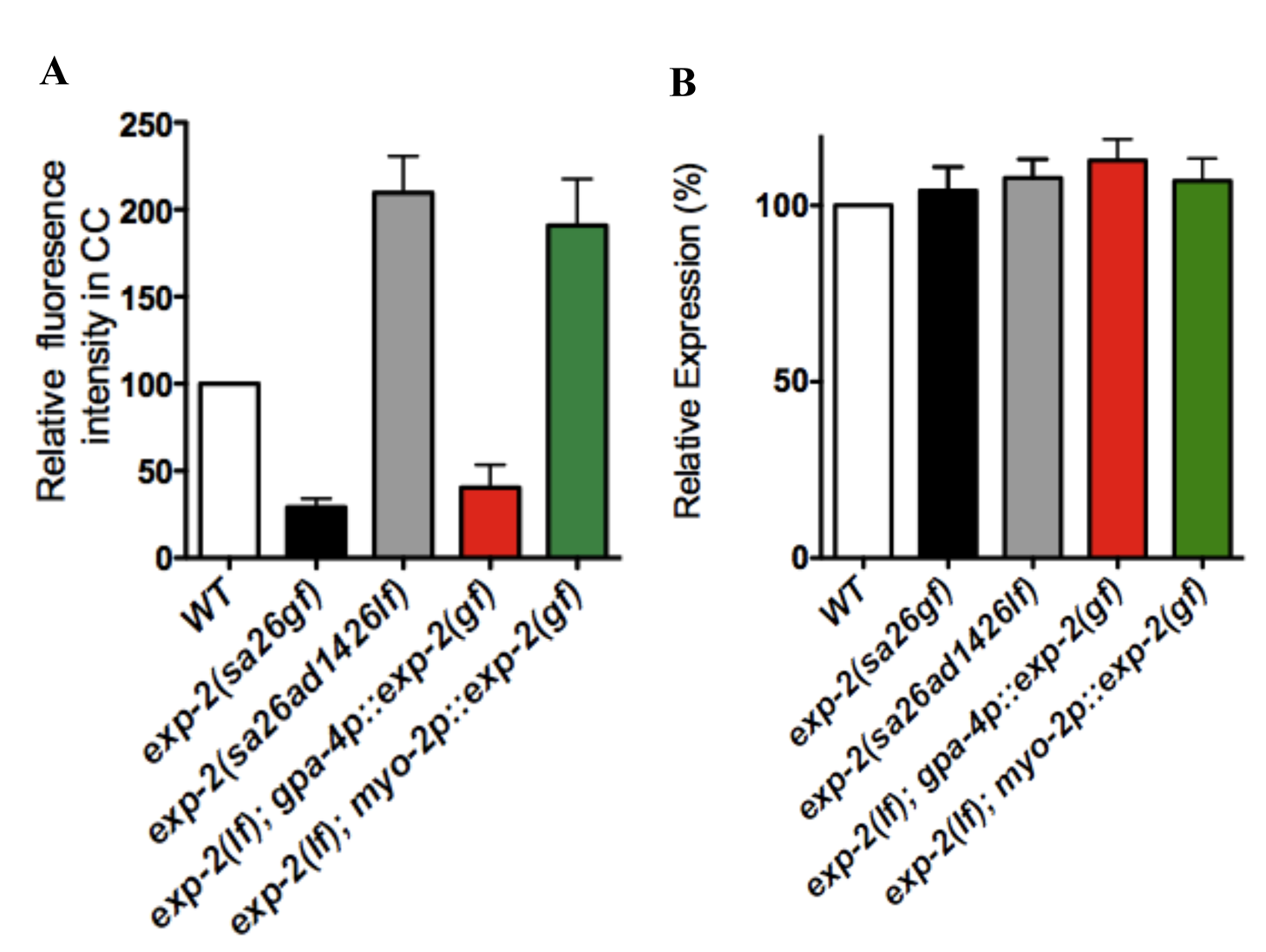

Supplement: Figure S5 — Coelomocyte DAF-28::GFP accumulation and daf-28 expression in exp-2 mutants. (A) Quantification of coelomocyte DAF-28::GFP in wild type worms, loss-of-function exp-2(sa26ad1426) and gain-of-function exp-2(sa26) mutants (p<0.001), and worms expressing exp-2(sa26) in ASI-specific (red bar, p>0.05 compared to exp-2(sa26) mutant) or pharyngeal-specific fashion in the exp-2(sa26ad1426) mutant background (green bar, p = 0.7966 compared to exp-2(sa26ad1426) mutant). (B) qPCR quantification of daf-28 mRNA levels in wild-type, loss-of-function exp-2(sa26ad1426), and gain-of-function exp-2(sa26) worms (p>0.05). Red and green bars represent mRNA levels in worms expressing exp-2 in ASI-specific or pharyngeal-specific fashion, respectively. Error bars indicate SEM. (TIF) [file pgen.1002519.s005.tif]

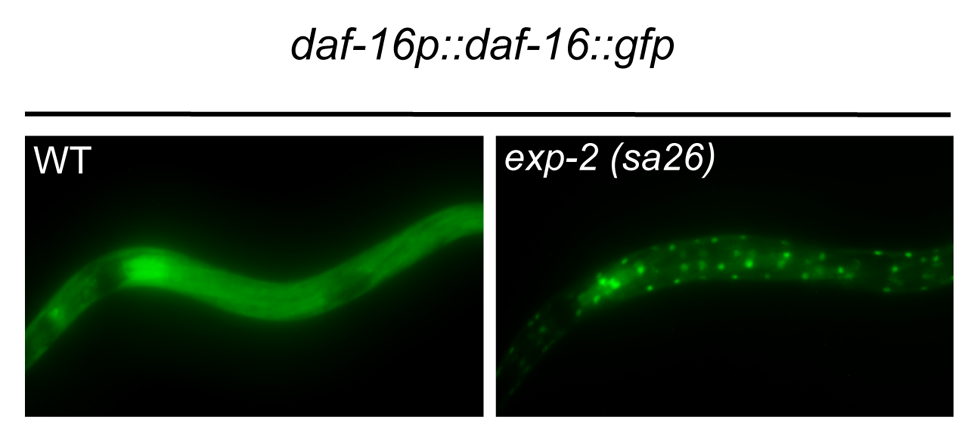

Supplement: Figure S6 — DAF-16::GFP exhibits nuclear localization in the gain-of-function exp-2(sa26) mutant. (TIF) [file pgen.1002519.s006.tif]
