# Supplementary material for: Repression of a Potassium Channel by Nuclear Hormone Receptor and TGF-β Signaling Modulates Insulin Signaling in Caenorhabditis elegans
Source: PLoS Genet. 2012 Feb 16;8(2):e1002519. doi: 10.1371/journal.pgen.1002519 (PMC3280960; doi:10.1371/journal.pgen.1002519)
Supplement: Table S1 — Life span phenotype described in the text. (DOC) [file pgen.1002519.s007.doc]

| Table S1. Life span phenotype described in the text | | |
| --- | --- | --- |
| Genotype | Mean life span ± s.e.m (N) | p-values |
| N2 | 15.2 ± 1.4 (195) | na |
| *daf-8(m85)*^a^ | 14.6 ± 0.8 (197) | 0.7692 |
| *nhr-69 (ok1926)*^a^ | 17.3 ± 1.3 (192) | 0.0276 |
| *m85 ok1926 a* | 21.5 ± 1.7 (188) | < 0.001 |
| *mIs39[nhr-69p::nhr-69::gfp]*^a^ | 11.8 ± 0.6 (193) | < 0.001 |
| *daf-2 (e1370)* | 33.6 ± 2.7 (183) | na |
| *exp-2(sa26ad1426)*^a^ | 9.4 ± 0.6 (176) | 0.0116 |
| *e1370; sa26ad1426*^b^ | 32.8 ± 1.7 (184) | 0.7606 |
| *m85 ok1926; sa26ad1426*^c^ | 10.8 ± 2.6 (182) | 0.7597 |
| **Life span in Fig. 6G** | | |
| N2 | 14.5 ± 0.7 (188) | na |
| *exp-2(sa26ad1426)*^d^ | 9.7 ± 1.1 (180) | 0.0104 |
| *exp-2(sa26ad1426); gpa-4p::exp-2*^e,f^ | 14.3 ± 1.6 (186) | 0.9181^e^, 0.0139^f^ |
| *exp-2(sa26ad1426); myo-2p::exp-2*^g,h^ | 8.7 ± 0.6 (191) | 0.0268^g^, 0.8326^h^ |
| Animals were maintained at 15^o^C. Life span experiments were carried out at 25^o^C. Age synchronized L4 animals were put on 2′fluoro-5′deoxyuridine (FUDR) (40 μM) containing plates to prevent progeny production. N, number of animals.  ^a^ Compared to N2  ^b^ Compared to *daf-2(e1370)*  ^c^Compared to *exp-2(sa26ad1426)*  ^d^Compared to N2  ^e^Compared to N2  ^f^Compared to *exp-2(sa26ad1426)*  ^g^Compared to N2  ^h^Compared to *exp-2(sa26ad1426)* | | |
