# Supplementary material for: Repression of a Potassium Channel by Nuclear Hormone Receptor and TGF-β Signaling Modulates Insulin Signaling in Caenorhabditis elegans
Source: PLoS Genet. 2012 Feb 16;8(2):e1002519. doi: 10.1371/journal.pgen.1002519 (PMC3280960; doi:10.1371/journal.pgen.1002519)
Supplement: Table S2 — PCR Primers described in the text. (DOC) [file pgen.1002519.s008.doc]

| Table S2 PCR Primers described in the text | |
| --- | --- |
| Name | Sequence |
| *nhr-69* | 5’-TGA AGT CTA CAT GCT CGA CAT GCG-3’;  5’-AAT CAA CTG GTA AAC ACT ACG GGG C-3’ |
| *gst-10* | 5’-ACG AGC AAG AGG CAA CCT TCC-3’;  5’-GCC TCC GGG ATG GTT TTG TT-3’ |
| *sod-3* | 5’-GCT GCA ATC TAC TGC TCG CAC TGC TTC-3’;  5’-GGC AAA TCT CTC GCT GAT ATT CTT CCA G-3’ |
| *exp-2* | 5’-CGC TGA AAC CGC TGA ACC TTG-3’  5’-AAT CCG AAC AAC AAG GAA AGC CC-3’ |
| *gfp* | 5’-GCA TGA CTT TTT CAA GAG TGC CAT G-3’  5’-CCT TCA AAC TTG ACT TCA GCA CCT G-3’ |
| dh_295_L | 5'atgggagctccaccacgtatatgtgaagaaagaacttaggaattcgatatcaagc 3’ |
| dh_296_R | 5’gcttgatatcgaattcctaagttctttcttcacatatacgtggtggagctcccat 3’ |
| dh_312_L | 5’ aaaaaGCTAGCatggtcgaagaaatatgtcatatttg 3’ |
| dh_313_R | 5’ AAAAAAAAAAGGGCCCctatttgtatagttcatccatgcc 3’ |
| dh_299_L: | 5’ aaaaaaggtacctctgagctaaactttttttttaacatg 3’ |
| dh_300_R | 5’ AAAAAAGGATCCttgcatcagtccgacgcagcggg3’ |
